# CircCFL1 Promotes TNBC Stemness and Immunoescape via Deacetylation‐Mediated c‐Myc Deubiquitylation to Facilitate Mutant TP53 Transcription

**DOI:** 10.1002/advs.202404628

**Published:** 2024-07-09

**Authors:** Zekun Wang, Yaming Li, Jingwen Yang, Yuhan Sun, Yinqiao He, Yuping Wang, Yiran Liang, Xi Chen, Tong Chen, Dianwen Han, Ning Zhang, Bing Chen, Wenjing Zhao, Lijuan Wang, Dan Luo, Qifeng Yang

**Affiliations:** ^1^ Department of Breast Surgery Qilu Hospital of Shandong University Jinan Shandong 250012 P. R. China; ^2^ School of Basic Medicine Jining Medical College Jining Shandong 272067 P. R. China; ^3^ Pathology Tissue Bank Qilu Hospital of Shandong University Jinan Shandong 250012 P. R. China; ^4^ Research Institute of Breast Cancer Shandong University Jinan Shandong 250012 P. R. China

**Keywords:** circular RNA, immune escape, mutant TP53, stemness, TNBC

## Abstract

Triple‐negative breast cancer (TNBC) is the most malignant subtype of breast cancer. TP53, which has a mutation rate of ≈70%–80% in TNBC patients, plays oncogenic roles when mutated. However, whether circRNAs can exert their effects on TNBC through regulating mutant TP53 has not been well evaluated. In this study, circCFL1, which is highly expressed in TNBC cells and tissues and has prognostic potential is identified. Functionally, circCFL1 promoted the proliferation, metastasis and stemness of TNBC cells. Mechanistically, circCFL1 acted as a scaffold to enhance the interaction between HDAC1 and c‐Myc, further promoting the stability of c‐Myc via deacetylation‐mediated inhibition of K48‐linked ubiquitylation. Stably expressed c‐Myc further enhanced the expression of mutp53 in TNBC cells with TP53 mutations by directly binding to the promoter of TP53, which promoted the stemness of TNBC cells via activation of the p‐AKT/WIP/YAP/TAZ pathway. Moreover, circCFL1 can facilitate the immune escape of TNBC cells by promoting the expression of PD‐L1 and suppressing the antitumor immunity of CD8^+^ T cells. In conclusion, the results revealed that circCFL1 plays an oncogenic role by promoting the HDAC1/c‐Myc/mutp53 axis, which can serve as a potential diagnostic biomarker and therapeutic target for TNBC patients with TP53 mutations.

## Graphical abstract

CircCFL1 enhanced the interaction between HDAC1 and c‐Myc, promoting the stability of c‐Myc via deacetylation‐mediated inhibition of K48‐linked ubiquitylation. The stabilized c‐Myc can further activate the transcription of mutant TP53, leading to the stemness and immunoescape of TNBC cells with TP53 mutations. The findings reveal that circCFL1 is a potential prognostic indicator and therapeutic target for TP53‐mutated TNBC patients.

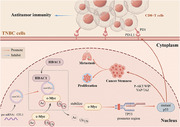

## 1 Introduction

Breast cancer remains the most common female malignancy worldwide, threatening the health of females.^[^ ^1^ ^]^ As the most malignant subtype, triple‐negative breast cancer (TNBC) accounts for 15%–20% of all breast tumors.^[^ ^2^ ^]^ TNBC is an immunohistochemistry (IHC)‐defined subtype of breast cancer that lacks the expression of estrogen receptor (ER) or progesterone receptor (PR) and exhibits human HER2 amplification, rapid proliferation, early metastasis and so on.^[^ ^3^, ^4^, ^5^ ^]^ Owing to the absence of the receptors mentioned above, conventional chemotherapy (CT) and radiotherapy (RT) endocrine therapy cannot be replaced by endocrine therapy for TNBC patients.^[^ ^6^ ^]^ Consequently, there is an urgent need to elucidate the molecular mechanisms and identify novel targets for TNBC progression.

Cancer is a complex, multifactorial and multistage disease caused by both endogenous and exogenous factors, such as environmental stimulation, metabolic changes, and genetic mutations.^[^ ^7^, ^8^ ^]^ Genetic mutations lead to the production of abnormal proteins in almost all cancers, and accumulating research has revealed that mutations in different genes could lead to different functions.^[^ ^9^ ^]^ For instance, BRCA‐mutant cells harbor DNA repair defects,^[^ ^10^ ^]^ and PIK3CA mutations regulate tumor immunogenicity.^[^ ^11^ ^]^ Remarkably, TP53 is regarded as the most frequently mutated gene in human tumors; although the fact that wild‐type TP53 is a well‐known tumor suppressor, some of its mutations can facilitate the malignant behaviors of cancers. Notably, TP53 mutations commonly occur in ≈70%−80% of TNBCs, leading to enhanced metastasis, stemness, chemoresistance, poor response to immunotherapy and so on.^[^ ^12^, ^13^, ^14^ ^]^ Several hot‐spot mutations of TP53 in TNBC have been identified and subtyped into contact mutations (R248Q, R273H, R280K) and structural mutations (Y220C, R249S, R282W),^[^ ^15^ ^]^ but the functions and regulatory mechanisms of mutp53 have not been clearly defined in TNBC.

Circular RNAs (circRNAs) are an emerging class of endogenous RNA transcripts characterized by covalent closed‐loop structures.^[^ ^13^ ^]^ In contrast to their linear counterparts, circRNAs are generated by backsplicing from precursor mRNAs lacking a 5′ m7G cap and 3′ poly(A) tail.^[^ ^16^ ^]^ Owing to their special circular structure, circRNAs are equipped with a longer half‐life, greater evolutionary conservation and greater resistance to RNase R digestion.^[^ ^17^ ^]^ Furthermore, circRNAs are highly abundant in eukaryotes, and some circRNAs are even more abundant than their linear counterparts,^[^ ^14^ ^]^ laying the foundation for their biological functions. It has been demonstrated that circRNAs exert their effects through different mechanisms, such as acting as miRNA sponges and interacting with proteins.^[^ ^14^ ^]^ Accumulating studies have emphasized that circRNAs play indispensable roles in carcinogenesis and cancer development.^[^ ^18^ ^]^ For instance, circ‐TRIO promotes the progression of TNBC by sponging miR‐432‐5p,^[^ ^19^ ^]^ and circRNA‐CREIT could facilitate the correlation between PKR and HACE1.^[^ ^20^ ^]^ However, the effects of circRNAs on TP53‐mutant TNBC have not been well evaluated, and identifying circRNAs that play regulatory roles in TNBC patients with TP53 mutations and elucidating the underlying mechanism might provide novel tailored targets for the treatment of TNBC patients with TP53 mutations.

In our study, we found that circCFL1 (hsa_circ_0000328), whose expression was strongly associated with the clinicopathological characteristics and prognosis of TNBC patients, was highly expressed in both TNBC cells and tissues. In vitro and in vivo experiments demonstrated that circCFL1 plays vital roles in promoting the proliferation, metastasis and stemness of TNBC cells. Mechanistically, circCFL1 could act as a scaffold to strengthen the interaction between Histone Deacetylase 1 (HDAC1) and c‐Myc, which enhanced the deacetylation‐mediated deubiquitylation of c‐Myc to stabilize its expression. Moreover, c‐Myc promoted mutp53 expression by directly binding to the promoter region of mutp53. Furthermore, circCFL1 promoted PD‐L1 expression in TNBC cells to inhibit the antitumor immunity of CD8^+^ T cells, which could be a synergetic target for anti‐PD‐L1 therapy. Taken together, our findings suggest a new mechanism of mutp53 regulation in TNBC and provide a novel prognostic biomarker and treatment target for TNBC patients.

## 2 Results

### 2.1 CircCFL1 Expression was Upregulated in TNBC and Associated with Poor Patient Prognosis

**Figure 1:**
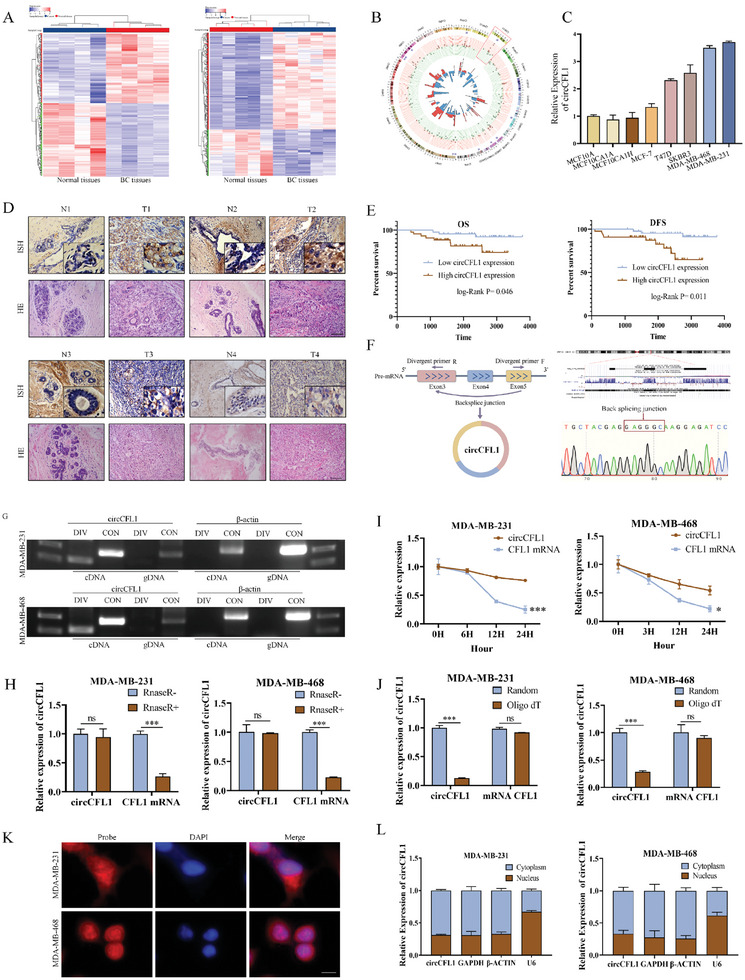
circCFL1 expression was upregulated in TNBC patients and associated with poor patient prognosis. A) Heatmaps of the significantly differentially expressed circRNAs between breast tissues and adjacent normal breast tissues (red represents upregulated circRNAs, and blue represents downregulated circRNAs). B) Circos plot indicating the differentially expressed circRNAs. The innermost circle and second circle show the results obtained from the above two circRNA arrays. The third circle (purple squares) represents the circRNAs that were both significantly differentially expressed in the two circRNA arrays. The outermost circle shows the chromosomal distribution of the circRNAs. C) qRT‒PCR was used to examine the expression of circCFL1 in breast cancer cell lines. D) ISH assays were conducted to determine the expression levels of circCFL1 in TNBC tissues and paired normal mammary tissues. Scale bars = 100 µm. E) Kaplan–Meier survival analysis of the prognostic value of circCFL1 for both OS and DFS (n = 44 patients in each group). F) Left panel, schematic illustration indicating that circCFL1 was circularized by exons 3–5 of human CFL1. Right panel, schematic diagram showing the genomic loci of circCFL1. The red arrow indicates the backsplicing site of circCFL1 confirmed by Sanger sequencing. G) Convergent and divergent primers were used for PCR, which suggested that circCFL1 could only be amplified from cDNA rather than from the gDNA of TNBC cells. H) Primers were used to amplify total RNA extracted from MDA‐MB‐231 and MDA‐MB‐468 cells treated with or without RNase R, and the expression levels of circCFL1 and CFL1 mRNA were verified by qRT‒PCR. I) Relative RNA levels of circCFL1 and CFL1 mRNA after actinomycin D treatment were measured by qRT‒PCR. J) Random 6‐mers primers or oligo (dT) primers were used for reverse transcription of cDNA, and the levels of circCFL1 and CFL1 mRNA reversed by those two different primers were analyzed by qRT‒PCR. K) Fluorescence in situ hybridization (FISH) with junction‐specific probes was utilized to determine the subcellular localization of circCFL1, and nuclei were stained with DAPI. Scale bars = 10 µm. L) Expression of circCFL1 in the cytoplasmic and nuclear fractions of RNAs extracted from TNBC cells. ns nonsignificant; *p < 0.05; **p < 0.01; ***p < 0.001.

To identify potential circRNAs associated with the progression of breast cancer, two circRNA arrays from the GEO database (GEO: 165884, GEO: 182471) were simultaneously analyzed to filter differentially expressed circRNAs between breast cancer tissues and paired adjacent normal tissues (**Figure** **1**A), and 19 upregulated circRNAs and 14 downregulated circRNAs were identified in breast cancer tissues compared with normal tissues by intersecting the results of both circRNA arrays (Figure 1B). Among them, circCFL1 (hsa_circ_0000328) was found to be overexpressed in TNBC cells and to be positively correlated with the aggressiveness of cancer cells, suggesting that circCFL1 might play crucial roles in the progression of TNBC (Figure 1C). Moreover, In situ hybridization (ISH) assays were also performed in clinical TNBC tissue samples, which further confirmed that circCFL1 was overexpressed in TNBC tissue samples compared with paired normal tissue samples (Figure 1D). To explore the prognostic value of circCFL1, the expression of circCFL1 in tumors of eighty‐eight TNBC patients were detected by qRT‐PCR assays, and were equally divided into two groups based on circCFL1 expression. As shown in Figure 1E, Kaplan–Meier plotter analysis revealed that high circCFL1 expression predicted poor prognosis in TNBC patients. The associations between circCFL1 expression and the clinicopathological characteristics of TNBC patients are shown in **Table** **1**, which indicates that circCFL1 expression is closely correlated with tumor size. Univariate and multivariate analyses were also conducted and demonstrated that circCFL1 expression was an independent prognostic factor for both Overall survivals (OS) (**Table** **2**) and Disease‐free survivals (DFS) (**Table** **3**). To summarize, our data suggested that circCFL1 might play a vital role in the tumorigenesis and progression of TNBC.

**Table 1:** Association between Clinicopathological variables and circCFL1 expression in TNBC patients.

		circCFL1 expressions		
Variable	Cases (n = 88)	Low	High	*P* value
Ag				
≤50	43	23	20	0.522
>50	45	21	24	
Histologic subtypes				
IDC	83	40	43	0.360
non‐IDC	5	4	1	
Histologic grade				1.000
G2	40	20	20	
G3	44	22	22	
Unknown	4	2	2	
Tumor size				0.041
≤2.5	59	34	25	
>2.5	29	10	19	
Lymph node status				0.509
Negative	55	29	26	
Positive	33	15	18	
Ki67 status				0.484
Low	9	6	3	
High	79	38	41	
Recurrence				0.118
No	76	41	35	
Yes	12	3	9	

**Table 2:** Univariate and multivariate analyses of prognostic factors (OS) for patients with TNBC.

Variable	Univariate analysis (OS)		Multivariate analysis (OS)	
	HR (95% CI)	*P* value	HR (95% CI)	*P* value
Age				
Age≤50	Reference	–		
Age>50	0.498 (0.150–1.659)	0.256		
Histological type				
IDC	Reference	–		
non‐IDC	0.041 (0.000–96.525)	0.420		
Histological grade				
G2	Reference	–		
G3	1.425 (0.452–4.492)	0.546		
Unknown	–	–		
Tumor size				
≤2 cm	Reference	–		
>2 cm	1.574(0.499–4.966)	0.439		
Lymph node status				
Negative	Reference	–	Reference	–
Positive	3.798 (1.143–12.621)	0.029	3.279 (0.981–10.963)	0.054
Ki67 status				
Low		–		
High	1.577 (0.200–12.100)	0.672		
circCFL1 expression				
Low	Reference	–	Reference	–
High	4.436 (1.187–16.583)	0.027	3.868 (1.028–14.552)	0.045

**Table 3:** Univariate analysis of prognostic factors (DFS) for patients with TNBC.

Variable	Univariate analysis (DFS)	
	HR (95% CI)	*P* value
Age		
≤45	Reference	–
Age>45	1.759 (0.380–8.148)	0.470
Histologic subtypes		
IDC	Reference	–
non‐IDC	0.043 (0.000–537.755)	0.514
Histological grade		
G2	Reference	–
G3	0.919 (0.281–3.013)	0.890
Unknown	–	–
Tumor size		
≤2 cm	Reference	–
>2 cm	1.554 (0.412–5.861)	0.515
Unknown	–	–
Lymph node status		
Negative	Reference	–
Positive	2.774 (0.844–9.119)	0.093
Ki67 status		
Low	Reference	–
High	0.627 (0.135–2.907)	0.551
CircCFL1 expression		
Low	Reference	–
High	5.615 (1.208–26.094)	0.028

CircCFL1 is derived from exons 3–5 of the CFL1 (Cofilin 1) gene located on human chromosome 11, and the endogenous existence of circCFL1 was confirmed by Sanger sequencing of the specific backsplicing site in TNBC cells (Figure 1F). To verify the circular characteristics of circCFL1, we designed divergent and convergent primers to amplify circCFL1 and linear CFL1 mRNA, respectively. cDNA and gDNA templates were then extracted from TNBC cell lines, and PCR assays revealed that circCFL1 could only be amplified from cDNA and not from gDNA, indicating that circCFL1 was a backsplicing product of pre‐mRNA (Figure 1G). Moreover, compared with CFL1, circCFL1 could withstand the digestion of RNase R (Figure 1H). Actinomycin D, an inhibitor of RNA synthesis, was also used to treat MDA‐MB‐231 and MDA‐MB‐468 cells, and the results suggested that circCFL1 was equipped with a much longer half‐life than CFL1 mRNA (Figure 1I). We also confirmed that circCFL1 but not CFL1 mRNA lacked a poly(A) tail, which was in accordance with the loop structure of circRNAs (Figure 1J). In addition, Fluorescence in situ hybridization (FISH) and nucleocytoplasmic separation assays were performed to determine the subcellular localization of circCFL1, which revealed that circCFL1 was located both in the cytoplasm and nucleus (Figure 1K,L). Therefore, circCFL1 is a circular RNA that is endogenously expressed in TNBC cells.

### 2.2 CircCFL1 Promoted the Proliferation, Migration and Invasion of TNBC Cells

**Figure 2:**
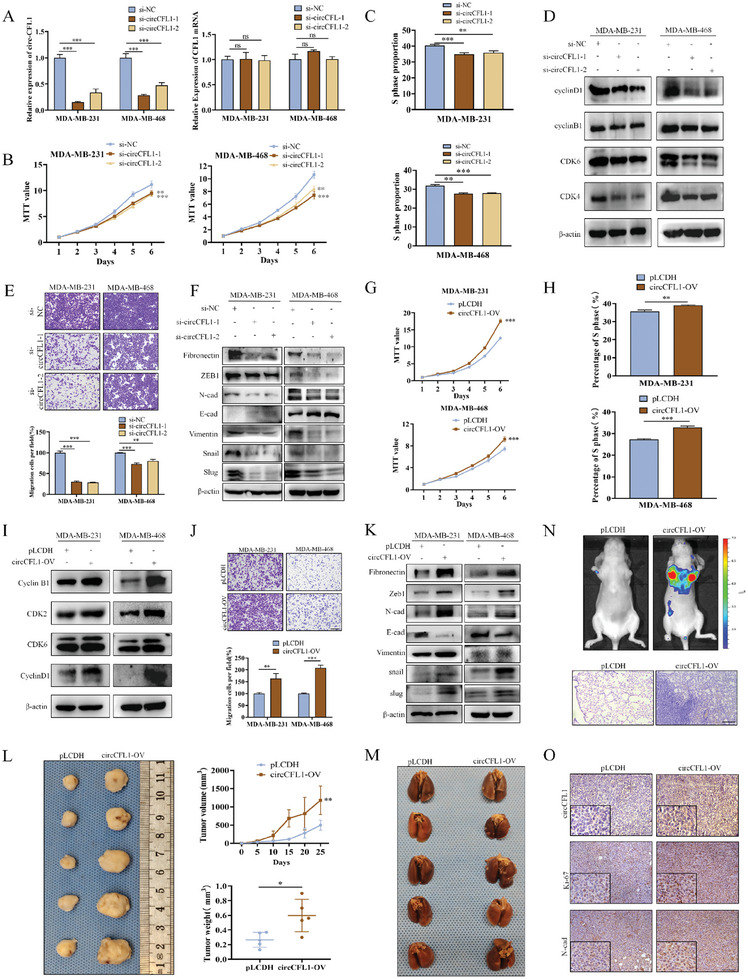
circCFL1 promoted the proliferation and metastasis of TNBC cells. A) The interference efficiencies of circCFL1‐targeting siRNAs on circCFL1 and CFL1 mRNA were verified by qRT‒PCR. B) MTT assays were performed to examine the proliferation rates of MDA‐MB‐231 and MDA‐MB‐468 cells after transfection with circCFL1 siRNAs. C) Statistical analysis of the flow cytometry data indicating the impact of circCFL1 knockdown on the proliferation of breast cancer cells. D) The expression levels of cell cycle‐related proteins were examined after circCFL1 knockdown. E) The migration abilities of MDA‐MB‐231 and MDA‐MB‐468 cells transfected with si‐circCFL1 were evaluated by Transwell assays. Scale bars = 200 µm. F) The expression levels of EMT signaling pathway‐related proteins were examined after circCFL1 knockdown. G) MTT assays were performed to determine the proliferation rates of MDA‐MB‐231 and MDA‐MB‐468 cells after transfection with circCFL1 overexpression vectors. H) Statistical analysis of the flow cytometry data suggested the impact of circCFL1 overexpression on the proliferation of breast cancer cells. I) The expression levels of cell cycle‐related proteins were verified in cells overexpressing circCFL1. J) The migration abilities of MDA‐MB‐231 and MDA‐MB‐468 cells transfected with circCFL1 overexpression vectors were evaluated by Transwell assays. Scale bars = 200 µm. K) The expression levels of EMT signaling pathway‐related proteins were altered after circCFL1 overexpression. L) Left panel, images of xenograft tumors obtained from BALB/c nude mice at the endpoint (n = 5 mice in each group). Right panel, growth curves and the volumes of xenograft tumors (n = 5 mice in each group). M) Images of lung metastatic nodules from BALB/c nude mice at the endpoint (n = 5 mice in each group). N) Animal in vivo imaging technology suggested tumor metastasis, and H&E staining confirmed tumor metastasis. Scale bars = 200 µm. O) ISH assay for circCFL1 and IHC staining for Ki67 and N‐cad in xenograft tumors. Scale bars = 100 µm. ns, nonsignificant; *p < 0.05; **p < 0.01; ***p < 0.001.

To evaluate the effects of circCFL1 on TNBC progression and metastasis, we first constructed two siRNAs that specifically target the back‐splicing junction of circCFL1 to knock down the expression of circCFL1 (Figure S1A, Supporting Information). As shown in **Figure** **2**A, both siRNAs efficiently knocked down the expression of circCFL1 but not CFL1 mRNA, which was further confirmed by FISH (Figure S1B, Supporting Information). Methyl thiazolyl tetrazolium (MTT), colony formation and 5‐Ethynyl‐2'‐deoxyuridine (EdU) assays confirmed that the proliferation of TNBC cells was inhibited after circCFL1 knockdown (Figure 2B; Figure S1C,D, Supporting Information). Moreover, flow cytometry and western blot assays confirmed that the cell cycle of both TNBC cell lines was blocked by circCFL1 inhibition (Figure 2C,D; Figure S1E, Supporting Information). We also evaluated whether circCFL1 knockdown could suppress the migration and invasion abilities of TNBC cells. Our Transwell and wound healing results confirmed that silencing circCFL1 markedly inhibited the migration and invasion of TNBC cells (Figure 2E; Figure S1F,G, Supporting Information). Furthermore, western blot assays also demonstrated that the Epithelial‐Mesenchymal Transition (EMT) pathway was significantly suppressed in both TNBC cell lines (Figure 2F). In addition, the effects of circCFL1 overexpression were also explored, and circCFL1 overexpression in TNBC cells was verified by qRT‒PCR and FISH assays (Figure S2A,B, Supporting Information). Our in vitro results demonstrated that circCFL1 overexpression promoted proliferation and cell cycle progression, as well as the migration, invasion and EMT of both TNBC cell lines (Figure 2G–K; Figure S2C–G, Supporting Information).

To evaluate the roles of circCFL1 in tumor progression and metastasis in vivo, MDA‐MB‐231 cells overexpressing circCFL1 were first seeded subcutaneously into female BALB/c nude mice along with negative controls to establish TNBC xenograft models. As shown in Figure 2L, the circCFL1‐overexpressing group exhibited increased tumor volume and weight, indicating that circCFL1 promoted TNBC growth in vivo. We also injected circCFL1‐overexpressing and control TNBC cells via the tail vein into female BALB/c nude mice to explore the influence of circCFL1 on metastasis, and the results demonstrated that the number of metastatic lung nodules in the circCFL1 group was markedly increased (Figure 2M,N; Figure S2H, Supporting Information). In vivo fluorescence imaging and Hematoxylin and eosin (HE) staining assays further confirmed our conclusions. ISH and Immunohistochemistry (IHC) were performed to detect circCFL1, Ki67 and N‐cad expression in circCFL1‐overexpressing and control tumors, which further indicated that circCFL1 could accelerate the proliferation and metastasis of TNBC in vivo (Figure 2O).

### 2.3 CircCFL1 Promoted the Stemness of TNBC Cells In Vitro and In Vivo

**Figure 3:**
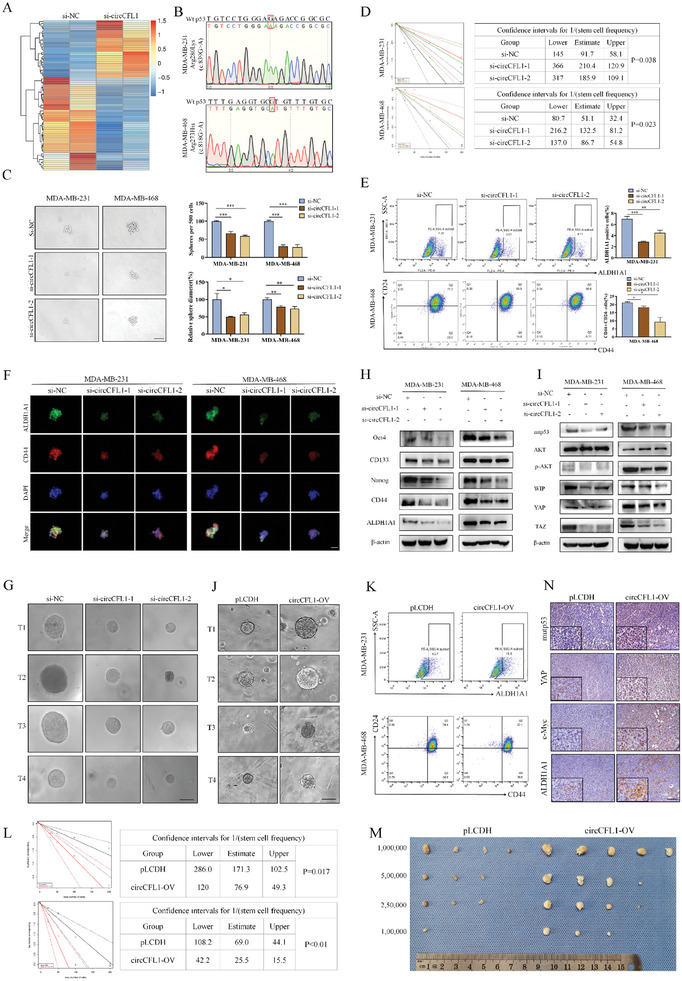
circCFL1 promotes the stemness of TNBC cells in vitro and in vivo. A) RNA sequencing analysis was performed in circCFL1‐knockdown TNBC cells. B) Sanger sequencing was used to identify the mutation site of TP53 in the MDA‐MB‐231 and MDA‐MB‐468 cell lines. Sphere formation assays C) and limiting dilution assays D) were used to evaluate the stemness properties of MDA‐MB‐231 and MDA‐MB‐468 cells after transfection with si‐circCFL1. Scale bars = 100 µm. E) Flow cytometry assays were performed to detect the effects of circCFL1 on the percentage of the ALDH1A1+ phenotype in MDA‐MB‐231 cells and the percentage of the CD44+/CD24‐ phenotype in MDA‐MB‐468 cells. F) IF staining assays were performed to evaluate the influence of circCFL1 siRNAs on the expression of stemness markers (CD44 and ALDH1A1) in TNBC cells. Scale bars = 50 µm. G) Patient‐derived organoid (PDO) models were generated after transfection with circCFL1 siRNAs or overexpression vectors. Scale bars = 50 µm. H) The expression of stemness‐associated proteins was altered after transfection with si‐circCFL1. I) Western blotting assays suggested that circCFL1 interference affected the expression of mutp53 and further suppressed the p‐AKT/WIP/YAP/TAZ signaling pathway in TNBC cells. J) Patient‐derived organoid (PDO) models were generated after transfection with a circCFL1 overexpression vector. Scale bars = 50 µm. K) Limiting dilution assays were used to determine the stemness properties of MDA‐MB‐231 and MDA‐MB‐468 cells after transfection with the circCFL1 overexpression vector. L) Flow cytometry assays were performed to detect the percentage of the ALDH1A1+ phenotype in MDA‐MB‐231 cells and the percentage of the CD44+/CD24‐ phenotype in MDA‐MB‐468 cells transfected with the circCFL1 overexpression vector. M) In vivo limiting dilution xenograft assays were conducted to evaluate the stemness of circCFL1‐overexpressing TNBC cells. N) IHC assays were utilized to detect the expression levels of mutp53, ALDH1A1, YAP, and c‐Myc. Scale bars = 100 µm. *p < 0.05; **p < 0.01; ***p < 0.001.

Since circCFL1 is regarded as a cancer‐promoting gene, we further aimed to identify the critical downstream effector correlated with the progression effects of circCFL1. RNA‐seq was first performed in circCFL1 knockdown and control cells to identify genes whose expression significantly changed, and KEGG enrichment analysis was subsequently performed, which suggested that circCFL1 knockdown might be correlated with the functions of the TP53 gene in TNBC cells (**Figure** **3**A; Figure S3A, Supporting Information). Since it has been reported that TP53 mutations occur in almost 70%−80% of TNBCs and that the mutations in MDA‐MB‐231 and MDA‐MB‐468 cells investigated in this study have been proven to encode oncogenic mutp53,^[^ ^12^ ^]^ we further hypothesized that circCFL1 might influence the role of mutp53 in TNBC cells. We also performed Sanger sequencing to examine the mutation status of TP53 in both TNBC cell lines, which verified that the MDA‐MB‐231 and MDA‐MB‐468 cells utilized in this study individually possessed the R280K and R273H mutation sites, which was in accordance with previous publications^[^ ^15^ ^]^ (Figure 3B).

It has been reported that one of the most important roles of mutp53 is affecting the stemness of tumor cells by activating the p‐AKT/WIP/YAP/TAZ axis,^[^ ^21^ ^]^ which was also significantly altered according to the KEGG analysis results shown in Figure S3A (Supporting Information). Thus, we evaluated whether circCFL1 could influence the stemness of TNBC cells by regulating mutp53 and the downstream p‐AKT/WIP/YAP/TAZ axis. First, tumor sphere formation and extreme limiting dilution analysis (ELDA) were performed, which demonstrated that circCFL1 knockdown reduced the stemness of TNBC cells (Figure 3C,D). Flow cytometry and IF assays of tumor stemness markers further confirmed the above results (Figure 3E,F). A previous study suggested that organoids are cell‐based in vitro models derived from stem cells that are equipped with stemness potential;^[^ ^22^, ^23^ ^]^ thus, we isolated cells from TNBC tissues to construct patient‐derived organoid (PDO) models to determine the correlation between circCFL1 and stemness. As shown in Figure 3G and Figure S3B (Supporting Information), our results showed that PDOs with circCFL1 knockdown had a slower growth rate, which further demonstrated that circCFL1 was correlated with the stemness of TNBC cells. Moreover, western blot assays were performed to examine the expression of stemness‐related markers and the mutp53/p‐AKT/WIP/YAP/TAZ axis, which demonstrated that circCFL1 knockdown inhibited the stemness of TNBC cells by regulating the mutp53‐regulated pathway (Figure 3H,I). The in vitro effects of circCFL1 overexpression on TNBC stemness were also detected and were consistent with the above findings (Figure 3J–L; Figure S3C–H, Supporting Information).

Subsequently, to evaluate the regulatory effects of circCFL1 on the stemness of TNBC cells in vivo, we performed xenograft experiments using limiting dilution assays of MDA‐MB‐231 cells. As shown in Figure 3M and Figure S3I (Supporting Information), our results indicated that the mice injected with circCFL1‐overexpressing TNBC cells had a dramatically increased incidence of tumor initiation compared to the control group. Moreover, stemness‐related proteins, including mutp53, YAP c‐Myc and ALDH1A1, were also detected by IHC in tumors derived from mice, which further confirmed that circCFL1 promoted the stemness of TNBC cells in vivo (Figure 3N). In conclusion, our in vitro and in vivo results demonstrated that circCFL1 enhanced the stemness of TNBC cells by affecting mutp53 and the downstream p‐AKT/WIP/YAP/TAZ axis.

### 2.4 CircCFL1 Directly Interacted with HDAC1

**Figure 4:**
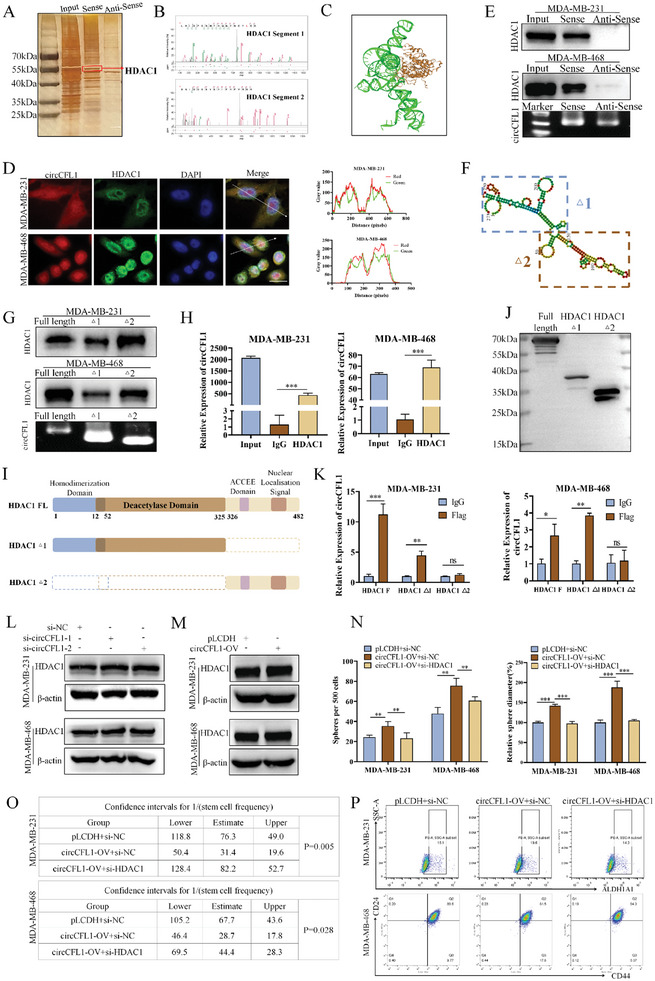
CircCFL1 directly interacted with HDAC1. A) Sliver staining image illustrating the enriched proteins precipitated by the RNA pull‐down assays of circCFL1. B) Representative segments of HDAC1 proteins identified by mass spectrometry (MS). C) Graphical representation of the molecular docking between circCFL1 and the HDAC1 protein by NPDock. D) FISH and IF assays were used to detect the subcellular localization of circCFL1 and HDAC1 (left). Colocalization analysis of circCFL1 and HDAC1 was conducted by ImageJ (right). Scale bars = 20 µm. E) Western blotting assays were used to verify the interaction between circCFL1 and HDAC1 in MDA‐MB‐231 and MDA‐MB‐468 cells. The effects of biotin‐labeled sense and antisense RNAs reverse transcribed from circCFL1 were examined by RNA blotting. F) The secondary structure of circCFL1 was predicted by the online tool RNAfold, and circCFL1 was divided into two truncations. The purple frame indicates △1, and the red frame indicates △2 of circCFL1. G) Western blotting assays were used to verify the specific truncations responsible for the interaction between HDAC1 and circCFL1. H) RIP assays confirmed the interaction between HDAC1 and circCFL1. I) Schematic diagram of full‐length and truncated HDAC1. J) Western blotting analysis was used to verify the expression efficiencies of full‐length and truncated HDAC1. K) RIP assays confirmed the correlation between HDAC1 truncations and circCFL1. Western blotting suggested that neither interference L) nor overexpression M) of circCFL1 affected the expression of HDAC1 at the protein level. Sphere formation N) and limiting dilution O) assays suggested that interference with HDAC1 could reverse the increase in stemness properties induced by circCFL1 overexpression. P) Flow cytometry assays were performed to determine the percentages of ALDH1A1‐ and CD44+CD24‐ cells among MDA‐MB‐231 and MDA‐MB‐468 cells after cotransfection with circCFL1‐OV and si‐HDAC1. ns, nonsignificant; *p < 0.05; **p < 0.01; ***p < 0.001.

To clarify the potential molecular mechanisms underlying the impact of circCFL1 on TNBC progression and stemness, an RNA pull‐down assay was first performed to identify proteins that interact with circCFL1. As shown in **Figure** **4**. A, We found that proteins of ≈55 kDa were significantly enriched with the circCFL1 probe, indicating that proteins with similar molecular weights may interact with circCFL1. Liquid chromatography‐tandem mass spectrometry (LC‒MS) was further used to identify specific proteins. HDAC1, with a predicted molecular weight of 55.1 kDa, had the highest binding affinity for circCFL1 (Figure S4A, Supporting Information), and the identified peptide sequences of HDAC1 are shown in Figure 4B. NPDock was used to predict the molecular docking between circCFL1 and the HDAC1 protein, further indicating the potential physical interaction between the two molecules (Figure 4C).^[^ ^24^ ^]^

To verify the interaction between circCFL1 and HDAC1, FISH and IF were first utilized to examine the subcellular localization of both circCFL1 and HDAC1, which confirmed that circCFL1 was present in both the cytoplasm/nucleus of TNBC cells and that HDAC1 was predominantly localized in the nucleus, suggesting that circCFL1 and HDAC1 can bind to each other (Figure 4D). In addition, RNA pull‐down assays were repeated in TNBC cells, and the results were detected with a specific HDAC1 antibody, which confirmed the interaction between circCFL1 and HDAC1 (Figure 4E). Furthermore, to determine the specific regions of circCFL1 that are crucial for binding to HDAC1, we constructed two truncated isoforms of circCFL1 based on the stem‒loop structure of circCFL1 predicted by RNAfold (Figure 4F). As shown in Figure 4G, the RNA pull‐down results proved that both isoforms of circCFL1 could interact with HDAC1, especially in the second part. Moreover, RIP assays using an HDAC1 antibody further verified the interaction between HDAC1 and circCFL1 (Figure 4H). To determine the specific regions of HDAC1 that are responsible for its interaction with circCFL1, we constructed two truncated vectors based on the structure of HDAC1, and the expression efficiency of both vectors was first examined (Figures 4I,J). RIP assays were then performed in TNBC cells overexpressing full‐length and truncated HDAC1, which indicated that HDAC1 ∆1, but not HDAC1 ∆2, was responsible for the interaction between circCFL1 and HDAC1 (Figure 4K). Taken together, our results demonstrated that circCFL1 could directly interact with HDAC1.

Since the above results demonstrated that circCFL1 could directly bind to HDAC1, we further explored whether circCFL1 could regulate the expression of HDAC1. Interestingly, neither knockdown nor overexpression of circCFL1 altered HDAC1 expression at the RNA or protein level, indicating that circCFL1 might influence the functions but not the expression of HDAC1 (Figure 4L,M; Figure S4B, Supporting Information). To verify whether HDAC1 is responsible for the oncogenic effects of circCFL1, we first analyzed HDAC1 expression in TNBC and adjacent normal tissues from TCGA and METABRIC databases and found that HDAC1 was upregulated in TNBC tissues, suggesting that HDAC1 is an oncogene in TNBC (Figure S4C, Supporting Information). Furthermore, HDAC1 expression was knocked down in circCFL1‐overexpressing TNBC cells (Figure 4N), and the effects of HDAC1 siRNA on the oncogenic functions of circCFL1 were examined (Figure S4D,E, Supporting Information). As shown in Figure 4O,P and Figure S4F–L (Supporting Information), our in vitro results proved that silencing HDAC1 suppressed the proliferation, migration, invasion and stemness of TNBC cells enhanced by circCFL1. In conclusion, our results demonstrated that circCFL1 promoted the malignant behaviors of TNBC cells by influencing the biological functions of HDAC1 without affecting its expression.

### 2.5 CircCFL1 Served as a Scaffold to Enhance the Interaction between HDAC1 and c‐Myc in TNBC Cells

**Figure 5:**
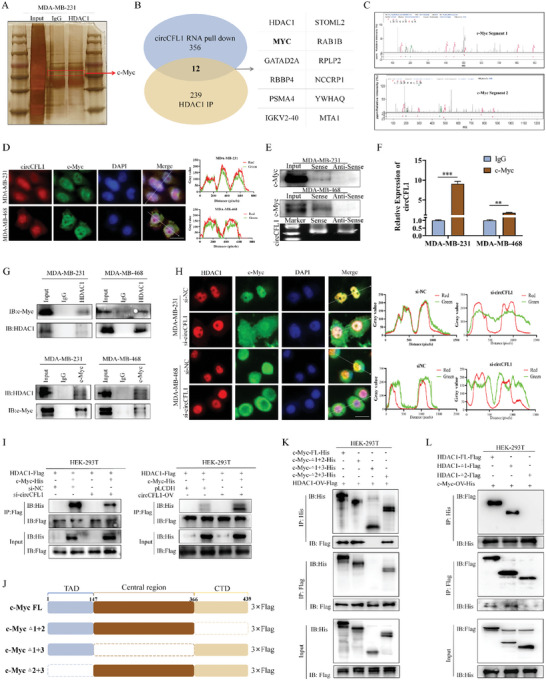
circCFL1 served as a scaffold to enhance the interaction between HDAC1 and c‐Myc in TNBC cells. A) Sliver staining image illustrating the enriched proteins precipitated in the immunoprecipitation assays of HDAC1. B) At the intersection of the circCFL1 RNA pull‐down and HDAC1 IP assays, 12 candidate proteins, including c‐Myc, were identified. C) Representative segments of c‐Myc proteins identified by mass spectrometry (MS). D) FISH and IF assays were performed to detect the colocalization of circCFL1 and c‐Myc. Scale bars = 20 µm. E) Western blotting assays were used to verify the interaction between circCFL1 and c‐Myc in MDA‐MB‐231 and MDA‐MB‐468 cells. F) A RIP assay was used to assess the interaction between c‐Myc and circCFL1. G) Co‐IP and western blot assays were conducted in MDA‐MB‐231 and MDA‐MB‐468 cells to verify the interaction between HDAC1 and c‐MYC. H) IF assays were used to detect changes in the subcellular localization of HDAC1 and c‐Myc following circCFL1 knockdown. Colocalization analysis for HDAC1 and c‐Myc was conducted by ImageJ. Scale bars = 20 µm. I) Western blotting was used to determine whether the overexpression or knockdown of circCFL1 affected the interaction between HDAC1 and c‐Myc. J) Schematic diagrams of full‐length and truncated c‐Myc. K) Western blot analysis showed that the central region of c‐Myc was essential for its interaction with HDAC1. L) Western blotting showed that the HDAC1‐∆1 region was responsible for its interaction with c‐Myc. ns nonsignificant; *p < 0.05; **p < 0.01; ***p < 0.001.

CircRNAs can act as scaffolds for specific proteins to modulate the degradation of downstream proteins.^[^ ^25^ ^]^ Consequently, we hypothesized that circCFL1 might act as a scaffold to link HDAC1 and other proteins since circCFL1 directly interacts with HDAC1 without affecting its expression to promote the progression and stemness of TNBC. To identify proteins that could interact with both HDAC1 and circCFL1, we further performed immunoprecipitation assays in TNBC cells with an HDAC1 antibody, and the enriched downstream proteins were analyzed by LC‒MS (**Figure** **5**A). As shown in Figure 5B, a total of 12 proteins were identified by intersecting the IP results and RNA pull‐down results shown in Figure 4A. Among the 12 proteins verified by IP and RNA pull‐down, c‐Myc, which has been reported to be closely correlated with mutp53 in cancers,^[^ ^26^ ^]^ was selected as the downstream cotarget of both HDAC1 and circCFL1, and the peptide sequence of c‐Myc identified by IP is shown in Figure 5C. First, to verify the interaction between circCFL1 and c‐Myc, IF assays were first performed, which revealed that circCFL1 and c‐Myc have similar subcellular localization in TNBC cells (Figure 5D). Moreover, the association between circCFL1 and c‐Myc was further verified by RNA pull‐down (Figure 5E) and RIP (Figure 5F) assays. Second, the interaction between HDAC1 and circCFL1 was also examined. As shown in Figure 5G, HDAC1 and c‐Myc antibodies were used to perform IP assays, and the c‐Myc and HDAC1 proteins were both enriched in TNBC cells, confirming the interaction between HDAC1 and c‐Myc, the results of which were also exogenously verified in HEK‐293T cells (Figure S5A, Supporting Information). To verify whether circCFL1 could function as a scaffold to enhance the interaction between HDAC1 and c‐Myc, IF assays were first performed to detect the subcellular locations of HDAC1 and c‐Myc after transfection with circCFL1 siRNA. As shown in Figure 5H, under normal conditions, both HDAC1 and c‐Myc were predominantly localized in the nucleus, while some c‐Myc translocated to the cytoplasm when circCFL1 was knocked down, suggesting the scaffold potential of circCFL1. Moreover, IP assays were performed to analyze the association between HDAC1 and c‐Myc under circCFL1 knockdown or overexpression conditions, which confirmed that the interaction between HDAC1 and c‐Myc could be strongly regulated by circCFL1 (Figure 5I).

We further explored the specific domains responsible for the interactions between HDAC1 and c‐Myc. Three truncated vectors were first constructed according to the structural domains of c‐Myc (Figure 5J). Full‐length and truncated c‐Myc vectors together with HDAC1 overexpression vectors were transfected into 293T cells, and IP assays suggested that ∆1+∆2 and ∆2+∆3, but not ∆1+3, could bind to the HDAC1 protein, indicating that the central region of c‐Myc was responsible for the correlation with HDAC1 (Figure 5K). Moreover, the HDAC1 truncation and full‐length vectors shown in Figure 4K together with the c‐Myc overexpression vector were also transfected into 293T cells, and the results revealed that only HDAC1 Δ1, which contains a deacetylase domain, could efficiently enrich the c‐Myc protein, indicating that HDAC1 might influence the acetylation level of c‐Myc (Figure 5L). In conclusion, our results demonstrated that circCFL1 could serve as a scaffold to enhance the interaction between HDAC1 and c‐Myc in TNBC cells.

### 2.6 The circCFL1/HDAC1 Axis Deacetylated c‐Myc at K148 to Protect c‐Myc from Degradation by Suppressing K48‐Linked Polyubiquitin

**Figure 6:**
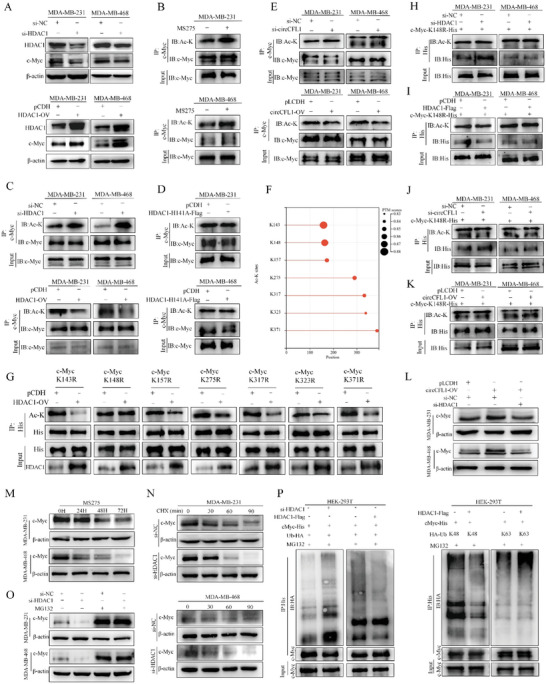
The circCFL1/HDAC1 axis deacetylated c‐MYC at K148 to protect c‐MYC from degradation by suppressing K48‐linked polyubiquitin. A) Western blotting assays suggested that either interference or overexpression of HDAC1 would impact the expression levels of c‐Myc. B) Western blotting assays indicated that treatment with MS275 impacted the acetylation level of c‐Myc in MDA‐MB‐231 and MDA‐MB‐468 cells. C) Interference or overexpression of HDAC1 influenced the acetylation level of c‐Myc. D) HDAC1 mutation, which abolished deacetylase activity, had no effect on the acetylation level of c‐Myc. E) Interference or overexpression of circCFL1 influenced the acetylation level of c‐Myc. F) Possible deacetylated sites on c‐Myc were predicted by MusiteDeep. G) The predicted deacetylated sites on c‐Myc were individually mutated (K‐R), and the effects of HDAC1 on the acetylation levels of the seven c‐Myc mutants were verified in 293T cells. The effects of HDAC1 H, I) and circCFL1 J,K) on the acetylation levels of c‐Myc with K148R were verified in both MDA‐MB‐231 and MDA‐MB‐468 cells. L) Cotransfection with circCFL1 overexpression vectors and HDAC1 siRNA inhibited the increase in the expression of c‐Myc induced by circCFL1 overexpression. M) MDA‐MB‐231 and MDA‐MB‐468 cells were treated with MS275 for 0, 24, 48, or 72 h, and the expression level of c‐Myc was examined. N) TNBC cells transfected with si‐HDAC1 were treated with 20 mg/mL CHX for 0, 30, 60, or 90 min, and the stability of c‐Myc was detected. O) MG132, a proteasome inhibitor, was utilized to treat TNBC cells at a concentration of 10 mM to verify the effects of HDAC1 on the proteasomal degradation of the c‐Myc protein. P) Co‐IP and western blotting were used to determine the effects of HDAC1 on the level of ubiquitinated c‐Myc. Q) Co‐IP and western blotting assays revealed that HDAC1 mediated c‐Myc degradation via K48‐linked ubiquitylation.

Since we demonstrated that circCFL1 promoted the interaction between HDAC1 and c‐Myc in TNBC cells, we further evaluated the effects of HDAC1 on c‐Myc expression. As shown in **Figure** **6**A and Figure S6A (Supporting Information), HDAC1 promoted the expression of c‐Myc at the protein level but not at the mRNA level, indicating that circCFL1 affects c‐Myc via posttranslational modifications (PTMs). As HDAC1 is a well‐known deacetylase, the effect of HDAC1 on c‐Myc acetylation was first explored in TNBC cells. By using MS275, an HDAC class I inhibitor, we found that the acetylation level of c‐Myc could be significantly altered (Figure 6B; Figure S6B, Supporting Information). Moreover, knockdown or overexpression of HDAC1 could enhance or suppress the acetylation of the c‐Myc protein, indicating that HDAC1 is involved in the acetylation of c‐Myc (Figure 6C; Figure S6C, Supporting Information). To further demonstrate that the deacetylation activity of HDAC1 regulates the acetylation level of c‐Myc, we constructed an H141A HDAC1 mutation vector, which has been reported to completely abolish the deacetylase activity of HDAC1.^[^ ^27^ ^]^ As shown in Figure 6D and Figure S6D (Supporting Information), the mutated version of HDAC1 had no effect on the acetylation level of c‐Myc, further confirming that the deacetylation activity of HDAC1 could regulate the acetylation level of c‐Myc. Similarly, circCFL1 was also silenced or overexpressed in TNBC cells, which confirmed that the acetylation level of c‐Myc could be regulated by the circCFL1/HDAC1 axis (Figure 6E).

To further explore the specific lysine on the c‐Myc protein that is regulated by HDAC1 in TNBC cells, MusiteDeep (https://www.musite.net/) was first utilized to predict lysine residues with acetylation potential, and 7 lysine residues (K143, K148, K157, K275, K317, K323 and K371) were predicted (Figure 6F). Lysine 7 was individually mutated to arginine to avoid acetylation (Figure S7E, Supporting Information), and the effects of HDAC1 on c‐Myc mutations were examined in 293T cells. HDAC1 overexpression had no significant effect on the K148R mutation of c‐Myc, indicating that K148 of c‐Myc is crucial for the regulation of HDAC1 (Figure 6G). Moreover, the effects of HDAC1 and circCFL1 on the acetylation level of c‐Myc at K148R were also examined in TNBC cells, confirming that the circCFL1/HDAC1 axis regulates c‐Myc acetylation at K148 (Figure 6H–K).

As shown in Figure 6L, the circCFL1/HDAC1 axis promoted the expression of c‐Myc at the protein level, and we next explored how the circCFL1/HDAC1 axis‐induced deacetylation of c‐Myc regulated its expression. Previous research indicated that acetylation might be associated with protein ubiquitination, and we hypothesized that circCFL1/HDAC1 axis‐induced c‐Myc deacetylation might stabilize the c‐Myc protein via deubiquitination.^[^ ^22^ ^]^ TNBC cells were first treated with MS275, and the results showed that a high acetylation level of c‐Myc led to inhibition of c‐Myc expression, suggesting that acetylation of c‐Myc might be crucial for its stabilization. (Figure 6M). Cycloheximide (CHX) treatment of TNBC cells indicated that knockdown of HDAC1 could accelerate the degradation of the c‐Myc protein, further suggesting that HDAC1‐induced deacetylation of c‐Myc could stabilize its expression (Figure 6N; Figure S6H, Supporting Information). Moreover, MG132, an inhibitor of the proteasome, was used to treat TNBC cells, and the results demonstrated that HDAC1 knockdown‐induced c‐Myc degradation was correlated with the ubiquitin‒proteasome system (Figure 6O). We thus detected the ubiquitination level of c‐Myc in TNBC cells treated with MS275, which proved that inhibition of deacetylase activity enhanced the ubiquitination level of c‐Myc (Figure S6I, Supporting Information). Knockdown or overexpression of HDAC1 and circCFL1 also demonstrated that the circCFL1/HDAC1 axis suppressed the ubiquitination of c‐Myc (Figure 6P; Figure S6J, Supporting Information). Moreover, the H141A mutation of HDAC1 had no effect on the level of ubiquitinated c‐Myc (Figure S6K, Supporting Information). We also explored whether HDAC1 regulates c‐Myc expression via K48‐ or K63‐linked polyubiquitin chains, as both are regarded as two types of well‐characterized polyubiquitin linkages in mammalian cells.^[^ ^28^ ^]^ As shown in Figure 6Q, the K48‐linked polyubiquitin level of c‐Myc was decreased after the overexpression of HDAC1 but not K63. In conclusion, our results demonstrated that the circCFL1/HDAC1 axis can deacetylate c‐Myc at K148 to protect c‐Myc from degradation by suppressing K48‐linked polyubiquitin.

We also evaluated whether c‐Myc was a functional downstream target of circCFL1. As shown in Figure S7 (Supporting Information), our results proved that interference with c‐Myc could inhibit the proliferation, migration, invasion and stemness promoted by circCFL1, proving that c‐Myc is crucial for the malignant behaviors of circCFL1 in TNBC.

### 2.7 The CircCFL1/HDAC1/c‐Myc Axis Promoted the Transcription of Mutant TP53 in TNBC Cells

**Figure 7:**
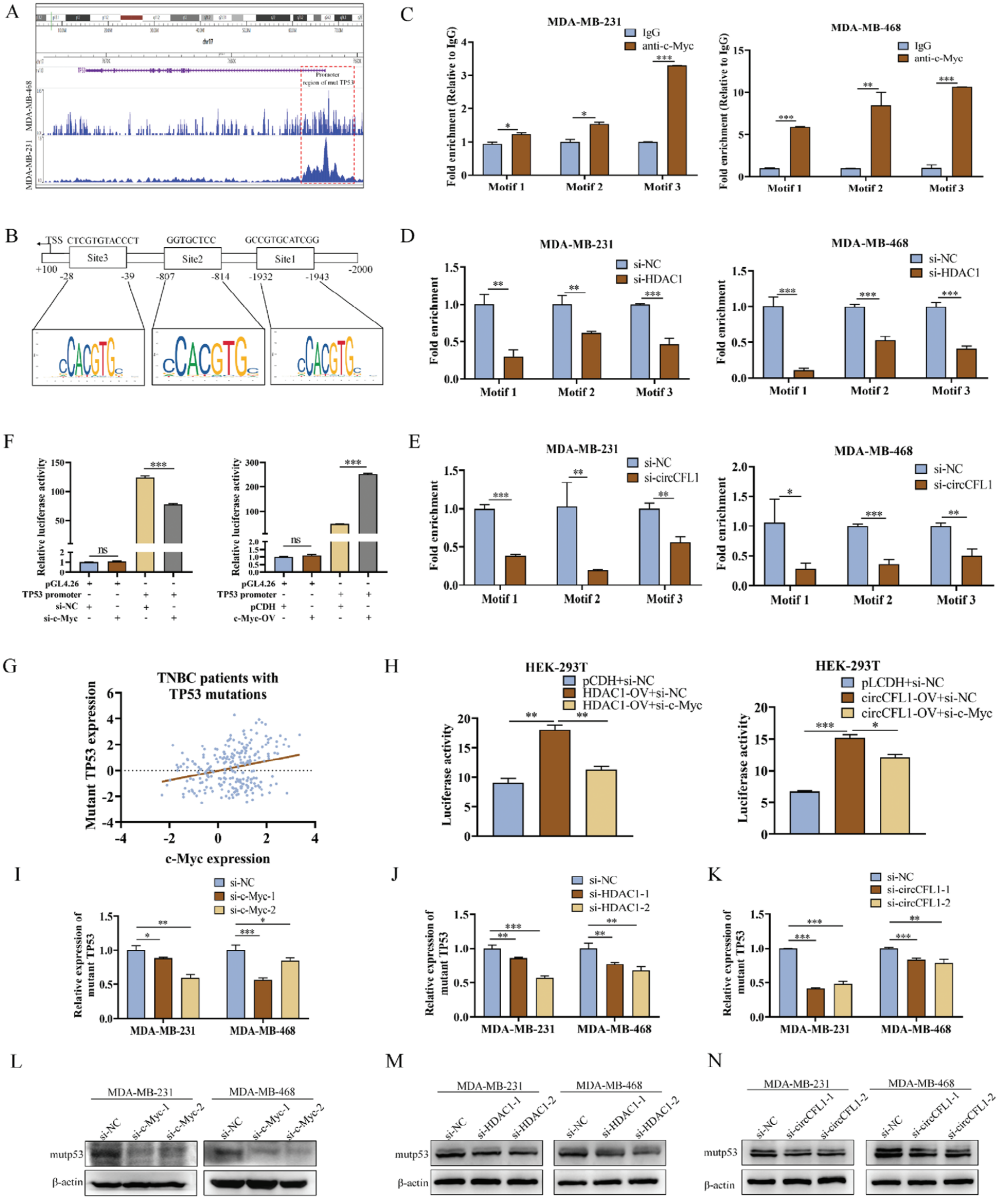
The CircCFL1/HDAC1/c‐Myc axis promoted the transcription of mutant TP53 in TNBC cells. A) ChIP results indicated that c‐Myc was enriched at the promoter of TP53 in TNBC cells with TP53 mutations. B) Schematic diagram showing the potential c‐Myc binding motif on the TP53 promoter predicted by JASPAR (https://jaspar.elixir.no/). C) ChIP results illustrated that c‐Myc could bind to all of the motifs on the TP53 promoter in TNBC cells. ChIP assays illustrated that both HDAC1 inhibition D) and circCFL1 inhibition E) decreased the binding capacity between c‐Myc and the TP53 promoter in TNBC cells with TP53 mutations. F) Dual‐luciferase reporter assays suggested that c‐Myc could enhance the transcriptional activity of TP53. G) The METABRIC database indicated a positive correlation between c‐Myc and TP53 expression in TNBC patients with TP53 mutations. H) Dual‐luciferase reporter assays suggested that the circCFL1/HDAC1/c‐Myc axis could regulate the transcriptional activity of TP53. I‒N) qRT‒PCR and western blot assays suggested that interference with the circCFL1/HDAC1/c‐Myc axis could regulate the expression of mutp53 at both the RNA and protein levels. *p < 0.05; **p < 0.01; ***p < 0.001.

Our previous results demonstrated that the functions of circCFL1 were correlated with mutant TP53 in TNBC cells and that c‐Myc has been reported to be a transcription factor of numerous oncogenes, including mutant TP53;^[^ ^29^ ^]^ thus, we examined whether the circCFL1/HDAC1/c‐Myc axis could promote mutant TP53 transcription in TNBC cells. As shown in **Figure** **7**A, based on Chip‐seq data from Cistrome (http://cistrome.org/), we found that c‐Myc could be enriched on the promoter of the TP53 gene in both MDA‐MB‐231 and MDA‐MB‐468 cells, indicating that c‐Myc has the potential to activate TP53 transcription. The promoter sequence of TP53 was further analyzed by JASPAR (https://jaspar.elixir.no/), and four potential binding sites of c‐Myc were predicted (Figure 7B). The ChIP results confirmed that all four motifs can bind to c‐Myc, which further confirmed the interaction between c‐Myc and the mutant TP53 promoter (Figure 7C; Figure S8A, Supporting Information). Moreover, the four c‐Myc binding motifs of TP53 were individually mutated and cloned and inserted into pGL4.26, which provided similar results (Figure S8B, Supporting Information). HDAC1 and circCFL1 were further silenced in TNBC cells, which confirmed that the circCFL1/HDAC1 axis was crucial for the binding of c‐Myc to the TP53 promoter in TNBC cells (Figure 7D,E).

To evaluate the effects of the circCFL1/HDAC1/c‐Myc axis on the transcriptional activity of mutant TP53 in TNBC cells, pGL4.26 vectors expressing the TP53 promoter were first cotransfected with c‐Myc siRNA and an overexpression vector, and the results proved that c‐Myc was crucial for the expression of TP53 (Figure 7F; Figure S8C–E, Supporting Information). Moreover, TP53‐mutant TNBC patients from the METABRIC database were selected, and a positive correlation between c‐Myc and mutant TP53 was also found (Figure 7G). Further dual‐luciferase assays confirmed that both circCFL1 and HDAC1 could also accelerate the transcription of TP53, which could be inhibited by c‐Myc siRNA, further providing evidence that the circCFL1/HDAC1/c‐Myc axis controls TP53 transcription (Figure 7H). Finally, the effects of siRNAs targeting c‐Myc, HDAC1 and circCFL1 on the expression of mutant TP53 in TNBC cells were examined, which further demonstrated that the circCFL1/HDAC1/c‐Myc axis regulated TP53 expression at both the mRNA and protein levels (Figure 7I–N).

We also verified whether knockdown of mutant TP53 could reverse the effects of circCFL1 on the proliferation, migration, invasion and stemness of TNC cells. The efficiency of TP53 siRNA was first verified in TNBC cells (Figure S9A, Supporting Information). Figure S9B (Supporting Information) shows that interference with mutp53 could reverse the enhanced proliferation of TNBC cells induced by circCFL1. Moreover, the inhibition of mutp53 decreased the migration and invasion abilities of TNBC cells overexpressing circCFL1 (Figure S9C,D, Supporting Information). Regarding the stemness of TNBC cells, mutp53 expression was also shown to be responsible for the tumorigenic effect of circCFL1 in TNBC cells (Figure S9E–H, Supporting Information). Furthermore, as shown in Figure S9I (Supporting Information), knockdown of mutp53 suppressed the PI3K/AKT/WIP/YAP/TAZ signaling pathway, whose activation has been proven to be crucial for the stemness of TP53‐mutant cancers. In conclusion, our results demonstrated that the circCFL1/HDAC1/c‐Myc axis enhanced the transcription of mutant TP53 to promote the progression and stemness of TNBC cells. In addition, 68 TNBC patients with TP53 mutations were equally divided into two groups according to the median expression of circCFL1 (n = 34 in each group), and TP53 mutated TNBC patients with high circCFL1 expression had poor outcomes. (Figure S10A,B, Supporting Information).

### 2.8 CircCFL1 Suppressed the Antitumor Immunity of CD8^+^ T Cells by Promoting PD‐L1 Expression in TNBC Cells with Mutant TP53

**Figure 8:**
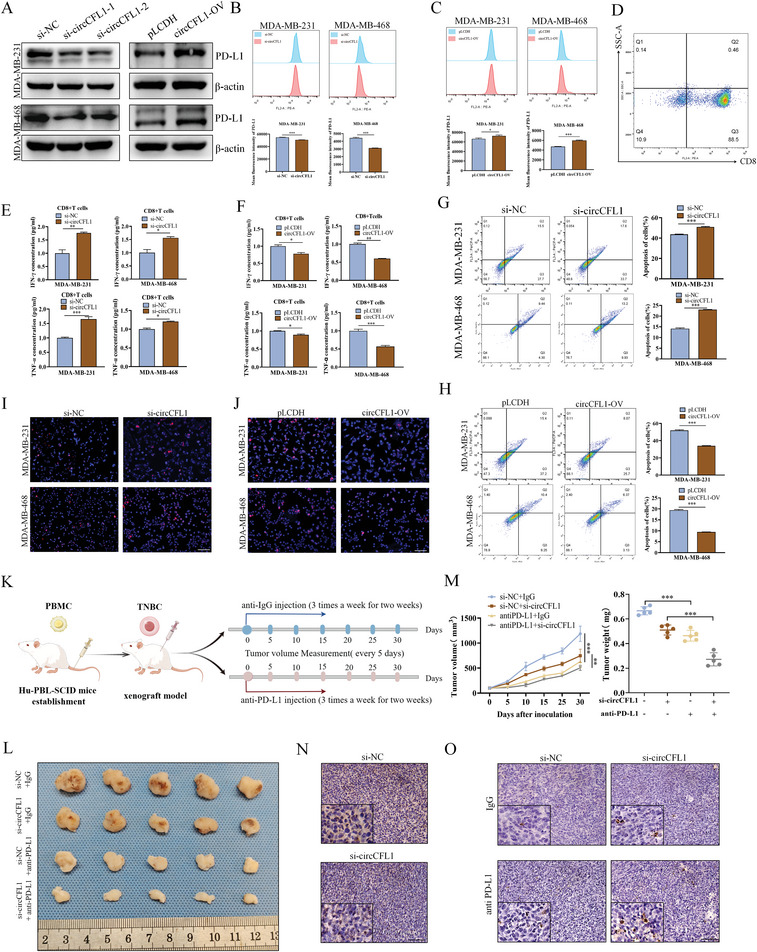
circCFL1 suppressed the antitumor immunity of CD8^+^ T cells by promoting PD‐L1 expression in TNBC cells with mutant TP53. A) Western blotting confirmed that circCFL1 expression was correlated with PD‐L1 expression. Flow cytometry assays illustrated that knockdown B) or overexpression C) of circCFL1 affected the expression of PD‐L1 in TNBC cells. D) Flow cytometry assays verified the extraction efficiency of CD8^+^ T cells from human PBMCs by using CD8 antibody. ELISA was performed to determine whether silencing E) or overexpressing F) circCFL1 in TNBC cells influenced the secretion of IFN‐γ and TNF‐α from cocultured CD8^+^ T cells. Flow cytometry G,H) and TUNEL I,J) assays illustrated that the CD8^+^ T‐cell‐induced apoptosis of MDA‐MB‐231 and MDA‐MB‐468 cells was impacted by the expression level of circCFL1. Scale bars = 100 µm. K) Schematic diagram showing the grouping and treatment procedure for our in vivo xenograft study. L) Images of xenograft tumors obtained from BALB/c nude mice at the endpoint (n = 5 mice in each group). M) Growth curves and volumes of xenograft tumors (n = 5 mice in each group). N) IHC assays were used to determine the PD‐L1 expression level after circCFL1 knockdown. Scale bars = 100 µm. O) TUNEL staining for apoptotic cells in xenograft tumors. Scale bars = 100 µm. *p < 0.05; **p < 0.01; ***p < 0.001.

The effects of mutp53 on tumor immunity have been reported previously, and mutp53 can upregulate PD‐L1 expression in tumor cells to promote immune escape; thus, we evaluated the role of circCFL1 in the antitumor immunity of CD8^+^ T cells to TNBC cells.^[^ ^30^ ^]^ First, qRT‒PCR, western blot, and flow cytometry assays were utilized to examine the expression of PD‐L1 in TNBC cells with circCFL1 knockdown or overexpression, which confirmed that PD‐L1 expression was positively correlated with the expression of circCFL1 (**Figure** **8**A‒C; Figure S10C,D, Supporting Information). Moreover, knockdown of mutp53 reversed the effects of circCFL1 on PD‐L1 expression, further indicating that circCFL1 could enhance PD‐L1 expression by regulating mutp53 and that circCFL1 might regulate the cytotoxicity of CD8^+^ T cells to TNBC cells (Figure S10E,F, Supporting Information). To verify our hypothesis, CD8^+^ T cells from human PBMCs were extracted, and the efficiency of the extraction was verified by flow cytometry (Figure 8D). CD8^+^ T cells were cocultured with TNBC cells with circCFL1 knockdown or overexpression, and ELISAs demonstrated that IFN‐γ and TNF‐α secreted by CD8^+^ T cells were positively correlated with circCFL1 expression in TNBC cells (Figure 8E,F). Moreover, in vitro assays demonstrated that circCFL1 inhibited CD8^+^ T‐cell‐induced TNBC cell apoptosis, further demonstrating that circCFL1 could suppress the antitumor effects of CD8^+^ T cells on TNBC cells (Figure 8G–J).

Based on the above in vitro results, we further explored whether circCFL1 is a potential target for anti‐PD‐L1 therapy in TNBC patients with mutant TP53. As shown in Figure 8K, a mouse model with reconstituted human immune system components was constructed. As shown in Figure 8L,M, knockdown of circCFL1 in TP53‐mutant TNBC cells sensitized TNBC tumors to anti‐PD‐L1 therapy, which was superior to monotherapy. Moreover, IHC assays demonstrated that circCFL1 interference downregulated PD‐L1 expression and enhanced anti‐PD‐L1 therapy‐induced TNBC cell apoptosis (Figure 8N,O). In conclusion, our results demonstrated that circCFL1 could be a potential target for improving the efficacy of anti‐PD‐L1 therapy in TNBC patients with TP53 mutations.

## 3 Discussion

TNBC is a heterogeneous and malignant disease that has relatively poor outcomes due to factors such as rapid proliferation, early metastasis and a deficiency of molecular targets for effective treatment.^[^ ^31^ ^]^ TNBC has been reported to be different from other subtypes in various aspects, such as gene expression and somatic mutations.^[^ ^32^ ^]^ TP53 is a commonly mutated gene in TNBC patients, with a mutation rate of ≈70%–80%. As a tumor suppressor, mutant TP53 functions as an oncogene to facilitate malignant behaviors of TNBC cells, such as proliferation, metastasis, stemness and immune escape.^[^ ^33^ ^]^ The expression and functions of mutant TP53 in cancers can be regulated by multiple factors. For example, TRIM21 can degrade mutp53,^[^ ^34^ ^]^ and LINC01088 decreases mutant TP53 expression and rescues the transcriptional activity of p53 by targeting the NPM1‐HDM2‐p53 axis.^[^ ^35^ ^]^ However, as a subtype of noncoding RNA with high evolutionary conservation and stability, the regulatory roles and mechanisms of circRNAs on mutant TP53 have not been fully elucidated in TNBC.

In this study, we identified circCFL1, which is upregulated in TNBC cells and tissues and is correlated with the clinicopathological characteristics and poor prognosis of TNBC patients, indicating that circCFL1 might be a functional circRNA in TNBC. In vitro and in vivo studies verified that knockdown of circCFL1 could inhibit the proliferation and metastasis of TNBC cells, and overexpression of circCLF1 had the opposite effect, which confirmed the vital roles of circCFL1 in TNBC. To fully understand the roles and potential mechanisms of circCFL1, RNA‐seq was first performed in TNBC cells with circCFL1 knockdown. Enrichment analysis indicated that the expression of circCFL1 might influence mutp53 in MDA‐MB‐231 cells, which harbor the R273H mutation of the p53 protein. Mutant TP53, generated from missense mutations of the wild‐type TP53 gene, is commonly accepted as an oncogene. p53 mutants can be classified into different types owing to their mutational sites and functions. For instance, R248Q, R273H and R280K were accepted as contact mutants since they occur in a residue directly involved in DNA binding without causing protein unfolding, while Y220C, R249S, R282W and the like were regarded as structural mutants for mutation‐induced destabilization resulting in structural distortion, unfolding, or aggregation.^[^ ^15^ ^]^ Stemness is one of the predominant properties of cancer cells regulated by mutp53, including R273H and R280K^[^ ^36^, ^37^ ^]^ For example, Solomon et al. reported that colorectal cancer cell lines expressing mutant TP53 exhibited increased populations of CD44‐, Lgr5‐ and ALDH‐positive cancer stem cells.^[^ ^38^ ^]^ Escoll et al. proposed that mutant TP53 promoted cancer stemness in breast cancer cells by activating WASP‐interacting protein (WIP) through the YAP/TAZ pathway, which is one of the most vital regulators of stemness in tumors.^[^ ^39^ ^]^ Cancer stem‐like cells (CSCs) are a type of cancer cell that possesses self‐renewal capacity and leads to tumor heterogeneity, aggressiveness and therapeutic resistance.^[^ ^40^ ^]^ A large number of studies have revealed that poor prognoses are closely associated with the acquired stemness properties of cancer cells.^[^ ^41^, ^42^, ^43^ ^]^ Stemness is also a substantial obstacle for the prognosis of TNBC patients, as TNBC is the most malignant type of breast cancer and is characterized by high stemness,^[^ ^44^ ^]^ which might result in a high percentage of recurrence and death in TNBC patients.^[^ ^45^ ^]^ Under these conditions, we further verified whether circCFL1 influenced the stemness of MDA‐MB‐231 and MDA‐MB‐468 cells, which harbor R273H and R280K of p53 with loss of normal DNA‐binding ability. We found that circCFL1 expression was positively correlated with stemness and mutp53 expression in TNBC cells. It has been reported that mutp53 can enhance cancer stemness by activating the p‐AKT/WIP/YAP/TAZ pathway, which is one of the most vital regulators of stemness in tumors. Our enrichment analysis also suggested that circCFL1 could participate in the PI3K/AKT pathway in TNBC cells, indicating that circCFL1 might affect TNBC stemness via the abovementioned pathway, which was verified by further experiments in this study.

Recently, the ability of circRNAs to bind proteins has attracted much attention, as circRNAs can act as scaffolds for promoting the binding of different proteins, further regulating the expression, functions and subcellular locations of downstream proteins.^[^ ^25^ ^]^ In the present study, HDAC1 was first identified as an interactor of circCFL1 by RNA pulldown, MS and RIP assays. As a member of the HDAC family, HDAC1 functions as an enzyme that catalyzes the lysine deacetylation of both histone and nonhistone proteins, exerting its primary role in diminishing the accessibility of transcription and further regulating the expression of some genes.^[^ ^46^, ^47^ ^]^ Moreover, previous studies indicated that HDAC1 was closely associated with tumor progression; for example, downregulation of HDAC1 inhibited the proliferation, migration and invasion of non‐small cell lung cancer cells,^[^ ^48^ ^]^ which indicated that HDAC1 could be a potential indicator for patient treatment. Further experiments in our study proved that circCFL1 did not directly influence the expression of HDAC1 but mainly interacted with the HDAC deacetylase domain of HDAC1, indicating that circCFL1 might affect the deacetylation function of HDAC1.^[^ ^49^ ^]^

To identify potential downstream targets regulated by both HDAC1 and circCFL1, IP and LC‒MS were performed using an HDAC1 antibody. The RNA pull‐down results of circCFL1 revealed that c‐Myc interacts with both circCFL1 and HDAC1, indicating that circCFL1 might act as a scaffold for HDAC1 and c‐Myc. Previous research has indicated that the oncogenic transcription factor c‐Myc has elevated and/or deregulated expression in more than 70% of all cancers.^[^ ^50^ ^]^ Belonging to the MYC family, the c‐Myc gene, located on human chromosome 8, encodes the transcription factor c‐Myc, which can regulate more than 15% of the human genome.^[^ ^51^, ^52^ ^]^ As a proto‐oncogene, c‐Myc has a high prevalence of deregulation and a causal role in tumor occurrence and progression;^[^ ^51^ ^]^ for instance, Li et al. proposed that c‐Myc could promote the metastasis of gastric cancer,^[^ ^53^ ^]^ and Calcagno et al. verified that c‐Myc deregulation could lead to gastric cancer.^[^ ^54^ ^]^

In this study, we demonstrated that the interaction between HDAC1 and c‐Myc could be enhanced by circCFL1. Previous studies have shown that the deacetylation of proteins is a key regulator of cancer stemness and progression.^[^ ^55^, ^56^ ^]^ Since HDAC1 is a well‐known deacetylase, we further evaluated whether the acetylation of c‐Myc could be regulated by HDAC1 and found that the deacetylation level of the K148 site of the c‐Myc sequence was predominantly regulated by HDAC1 and circCFL1. The deacetylation of proteins is widespread among eukaryotes, leading to changes in the structure, subcellular location and stabilization of proteins.^[^ ^57^, ^58^, ^59^, ^60^ ^]^ In our study, we found that both HDAC1 and circCFL1 could influence the expression of c‐Myc at the translational but not the transcriptional level, indicating that HDAC1 might cooperate with circCFL1 to affect the stabilization of the c‐Myc protein. Previously, studies have reported that acetylation and ubiquitination are often associated with each other with either positive or negative correlations; for example, C/EBPbeta could be regulated by SIRT2‐mediated deacetylation and deubiquitination; however, OTUD3 could be regulated by acetylation‐dependent deubiquitinase.^[^ ^60^, ^61^ ^]^ Consequently, we explored whether the above conclusion was also suitable for the regulation of c‐Myc in our study. The results confirmed that the enhanced interaction between HDAC1 and c‐Myc by circCFL1 led to the deacetylation of c‐Myc, further stabilizing c‐Myc expression by inhibiting K48‐mediated ubiquitylation, indicating that c‐Myc is regulated by deacetylation‐mediated deubiquitylation.

Since we demonstrated that the oncogenic role of circCFL1 was correlated with mutp53 in TNBC cells, we further explored how the circCFL1/HDAC1/c‐Myc axis influences mutp53 function. As a transcription factor, c‐Myc has been reported to regulate the transcription of numerous genes, including mutant TP53 in pancreatic cancer, lymphoma and so on,^[^ ^62^, ^63^ ^]^ but the effect of c‐Myc on mutant TP53 transcription in breast cancer has not been well studied. In our study, we demonstrated that c‐Myc could directly bind to the promoter and activate the transcription of mutant TP53 in TNBC cells, which could be regulated by both circCFL1 and HDAC1. It has been reported that mutp53 could also inhibit the degradation of the c‐Myc protein,^[^ ^64^ ^]^ indicating that enhanced mutp53 expression could further facilitate our circCFL1/HDAC1/c‐Myc axis and form positive feedback to promote the initiation and progression of TNBC with mutant TP53. In our study, multiple p53 TNBC cell lines, including MDA‐MB‐231 and MDA‐MB‐468, which individually harbor R273H and R280K mutations, respectively, were reported as contact mutants with impaired DNA binding functions,^[^ ^65^, ^66^ ^]^ which was in accordance with our results that the circCFL1/HDAC1/c‐Myc/mutp53 axis promoted progression and stemness in mutant TP53 TNBC.

Immune checkpoint blockade therapies (ICBs) blocking the interaction between programmed cell death protein 1 (PD‐1) and its ligand (PD‐L1) have shown immense benefits for the treatment of some cancers, including durable responses and prolonged survival.^[^ ^67^ ^]^ For instance, the anti‐PD‐1 monoclonal antibody pembrolizumab has been used to treat non‐small cell lung cancer^[^ ^68^ ^]^ and metastatic gastric cancer.^[^ ^69^ ^]^ However, although some patients with aggressive triple‐negative breast cancer (TNBC) are PD‐L1 positive (defined as 1% of tumor cells and/or tumor‐infiltrating immune cells expressing PD‐L1), they respond poorly to ICB therapy and exhibit resistance to anti‐PD‐1/PD‐L1 therapy.^[^ ^70^ ^]^ Therefore, there is an urgent need to improve the efficacy of ICB in TNBC patients. Previous studies proposed that tumor cells with mutant TP53 possess high expression of PD‐L1 at both the mRNA and protein levels, which could suppress the cytotoxic effects of T cells and protect tumors from effector‐immune responses.^[^ ^71^, ^72^, ^73^ ^]^ For instance, Liu et al. reported that mutant TP53 enhances the translational level of PD‐L1 via the PHLPP2/AKT pathway, and T. Alexander et al. suggested that mutant TP53 increases IFN‐ɣ‐induced PD‐L1 expression.^[^ ^72^, ^74^ ^]^ Hence, mtp53 might be a novel biomarker correlated with resistance to anti‐PD‐1/PD‐L1 therapy. In addition, Dario Zimmerli1 et al. illustrated that Myc could promote the immune suppression of TNBC, indicating that circCFL1 might play similar roles in regulating tumor immunity.^[^ ^75^ ^]^ As PD‐L1 mainly targets CD8^+^ T cells in the tumor microenvironment,^[^ ^76^ ^]^ we explored whether the expression of circCFL1 could regulate the expression level of PD‐L1 in mutp53 TNBC cells and CD8^+^ T‐cell‐induced TNBC cell death. Our results proved that circCFL1 could enhance PD‐L1 expression in both MDA‐MB‐231 and MDA‐MB‐468 cells, further inhibiting CD8^+^ T‐cellinduced TNBC cell death. Moreover, we evaluated the effects of circCFL1 shRNA on anti‐PD‐L1 therapy by in vivo experiments, which demonstrated that circCFL1 shRNA combined with anti‐PD‐L1 could significantly decrease tumor size. Our discovery demonstrated for the first time that circCFL1 is a tumor‐specific target for anti‐PD‐L1‐based therapy, providing a novel and effective treatment strategy for TNBC patients with mutant TP53.

## 4 Conclusion 

**Figure 9:**
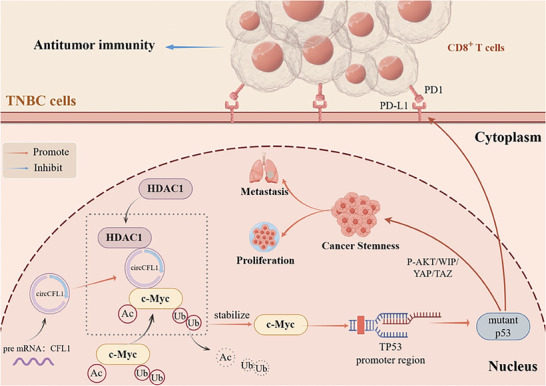
A schematic diagram of the underlying mechanism indicates that circCFL1 can promote the immune escape and stemness of TNBC cells by enhancing the activity of the HDAC1/c‐Myc/mutp53 axis.

In summary, our study revealed that circCFL1, whose expression was upregulated in TNBC tissues and associated with poor prognosis in TNBC patients, plays vital roles in promoting the progression and stemness of TNBC patients with TP53 mutations by regulating the expression of mutp53. Mechanistically, circCFL1 could act as a scaffold to enhance the interaction between HDAC1 and c‐Myc, further promoting the deacetylation‐mediated deubiquitylation of the c‐Myc protein to stabilize its expression. Moreover, c‐Myc promoted the accumulation of the mutp53 protein through transcriptional regulation, further facilitating stemness via the p‐AKT/WIP/YAP/TAZ signaling pathway. We also found that circCFL1 inhibited CD8^+^ T‐cell‐mediated antitumor immunity by promoting PD‐L1 expression in TNBC cells with TP53 mutations. The newly identified circCFL1 broadens our insights into the underlying mechanisms of mutant TP53 regulation and represents a potential prognostic and therapeutic target for the treatment of TNBC patients with TP53 mutations (**Figure** **9**).

## 5 Experimental Section

### Ethics Statement and Human Tissue Samples

All of the experimental procedures in this research were approved by the Ethical Committee of Qilu Hospital of Shandong University and all patients participating in this study have provided written, informed consent for utilization of these clinical materials in research. Tissues were obtained from TNBC patients undergoing surgery in Qilu Hospital of Shandong University and immediately stored at −80 °C.

### Animal Experiments

For xenograft proliferation studies, MDA‐MB‐231 expressing pLCDH and circCFL1 cells (1 × 10^7^ cells) in 200 µL of PBS containing Matrigel (1:3, v/v) was injected subcutaneously into the left flank of 4 to 6‐week‐old BALB/c nude female mice (n = 5 for each group). Tumor growth rate was monitored by measuring tumor diameters every 5 days. At the endpoint, the mice were sacrificed, and the xenografted tumors were measured and weighed. For pulmonary metastasis studies, 5 × 10^5^ cells with circCFL1 overexpression or not were injected into the lateral tail veins of 4‐ to 6‐week‐old BALB/c nude female mice (five mice per group). At the endpoint, all of the mice were killed under anesthesia. The lungs were collected and fixed in 10% formalin. For anti‐PD1 therapy experiments, xenograft experiments were performed in Hu‐PBL‐SCID mice. Briefly, 1.0 × 10^7^ PBMC cells extracted from healthy donor were intravenous injected into mice to construct Hu‐PBL‐SCID mice. Then, MDA‐MB‐231 cells (1 × 10^7^cells cells per mouse) stably expressing sh‐circCFL1 or sh‐NC were subcutaneously injected into the Hu‐PBL‐SCID mice. When tumors reached a size of ≈100 mm^3^, mice were randomly assigned into different groups with anti‐PD‐L1 or IgG, three times a week for 2 weeks (n = 5 for each group). Every five days, the tumor size was measured, with Day 1 being the day the mice received their first dose. The formula used to compute the tumor volume was volume = (length × width^2^)/2. After being surgically removed, the tumor specimens were fixed, embedded in paraffin, and sectioned. Hematoxylin and eosin (H&E) and immunohistochemistry (IHC) staining were performed on the sections.

### Statistical Analysis

Statistical analysis was conducted by GraphPad Prism 8.0. All data were represented as mean ± standard deviation (SD) and are derived from a minimum of three independent experiments. Student's *t*‐test or one‐way ANOVA was utilized to evaluate the relationship between parametric variables. Chi‐square test was applied to analyze the relationships between nonparametric variables. Kaplan–Meier analysis was used to analyze the survival differences. *P* < 0.05 was regarded statistically significant.

## Conflict of Interest

The authors declare no conflict of interest.

## Author Contributions

Z.W. and Y.L. contributed equally to this work. Q.F.Y., Z.K.W., and Y.M.L. conceived and designed the study. Z.K.W., Y.M.L., J.W.Y., Y.H.S., Y.Q.H., and Y.P.W. performed the experiments; Y.R.L., T.C., D.W.H., N.Z., and L.J.W. collected clinical samples; Y.M.L., X.C., Y.R.L., B.C., D.L., and W.J.Z. analyzed the data; Z.K.W. and Y.M.L. wrote the paper; Y.M.L. and Q.F.Y. revised the paper. All authors read and approved the final manuscript.

## Ethics approval and consent to participate

This project was approved by the Ethical Committee on Scientific Research of Shandong University Qilu Hospital.

## Supporting information

Supporting Information

## Data Availability

Research data are not shared.

## References

[1] H. Sung , J. Ferlay , R. L. Siegel , M. Laversanne , I. Soerjomataram , A. Jemal , F. Bray , CA Cancer J. Clin. 2021, 71, 209. 33538338 10.3322/caac.21660

[2] S. Haque , K. Cook , G. Sahay , C. Sun , Pharmaceutics 2021, 13, 1694. 34683988 10.3390/pharmaceutics13101694 PMC8537780

[3] C. Liao , Y. Zhang , C. Fan , L. E. Herring , J. Liu , J. W. Locasale , M. Takada , J. Zhou , G. Zurlo , L. Hu , J. M. Simon , T. S. Ptacek , V. G. Andrianov , E. Loza , Y. Peng , H. Yang , C. M. Perou , Q. Zhang , Cancer Discov. 2020, 10, 1706. 32690540 10.1158/2159-8290.CD-20-0288 PMC7642036

[4] S. Liu , M. van Dinther , S. C. Hagenaars , Y. Gu , T. B. Kuipers , H. Mei , M. C. Gomez‐Puerto , W. E. Mesker , P. ten Dijke , Int. J. Cancer 2023, 152, 2594. 36823950 10.1002/ijc.34483

[5] S. K. Yeo , J. L. Guan , Trends Cancer 2017, 3, 753. 29120751 10.1016/j.trecan.2017.09.001 PMC5802368

[6] H. A. Wahba , H. A. El‐Hadaad , Cancer Biol. Med. 2015, 12, 106. 26175926 10.7497/j.issn.2095-3941.2015.0030 PMC4493381

[7] B. C. Yoo , K. H. Kim , S. M. Woo , J. K. Myung , J. Proteomics 2018, 188, 97. 28821459 10.1016/j.jprot.2017.08.010

[8] E. M. Giatagana , A. Berdiaki , A. Tsatsakis , G. N. Tzanakakis , D. Nikitovic , Biomolecules 2021, 11. 10.3390/biom11091319 PMC8466546 34572532

[9] L. B. Alexandrov , S. Nik‐Zainal , D. C. Wedge , S. A. J. R. Aparicio , S. Behjati , A. V. Biankin , G. R. Bignell , N. Bolli , A. Borg , A. L. Børresen‐Dale , S. Boyault , B. Burkhardt , A. P. Butler , C. Caldas , H. R. Davies , C. Desmedt , R. Eils , J. E. Eyfjörd , J. A. Foekens , M. Greaves , F. Hosoda , B. Hutter , T. Ilicic , S. Imbeaud , M. Imielinski , N. Jäger , D. T. W. Jones , D. Jones , S. Knappskog , M. Kool , et al., Nature 2013, 500, 415.23945592

[10] H. Farmer , N. McCabe , C. J. Lord , A. N. J. Tutt , D. A. Johnson , T. B. Richardson , M. Santarosa , K. J. Dillon , I. Hickson , C. Knights , N. M. B. Martin , S. P. Jackson , G. C. M. Smith , A. Ashworth , Nature 2005, 434, 917. 15829967 10.1038/nature03445

[11] S. Choi , H. Kim , Y. J. Heo , S. Y. Kang , S. Ahn , J. Lee , K. M. Kim , J. Pathol. 2023, 260, 443. 37341658 10.1002/path.6134

[12] X.i Su , C. Feng , S. Wang , L. Shi , Q. Gu , H. Zhang , X. Lan , Y. Zhao , W. Qiang , M. Ji , P. Hou , Cell Death Differ. 2021, 28, 2450. 33742136 10.1038/s41418-021-00762-7 PMC8329294

[13] D. Ghatak , D. D. Ghosh , S. Roychoudhury , Front Oncol. 2020, 10, 604124. 33505918 10.3389/fonc.2020.604124 PMC7830093

[14] N. Sobhani , A. D'Angelo , X. Wang , K. H. Young , D. Generali , Y. Li , Int. J. Mol. Sci. 2020, 21, 4087. 32521648 10.3390/ijms21114087 PMC7312027

[15] A. Gomes , F. Trovão , B. Andrade Pinheiro , F. Freire , S. Gomes , C. Oliveira , L. Domingues , M. Romão , L. Saraiva , A. Carvalho , Int. J. Mol. Sci. 2018, 19, 1184. 29652801 10.3390/ijms19041184 PMC5979565

[16] W. R. Jeck , N. E. Sharpless , Nat. Biotechnol. 2014, 32, 453. 24811520 10.1038/nbt.2890 PMC4121655

[17] S. Memczak , M. Jens , A. Elefsinioti , F. Torti , J. Krueger , A. Rybak , L. Maier , S. D. Mackowiak , L. H. Gregersen , M. Munschauer , A. Loewer , U. Ziebold , M. Landthaler , C. Kocks , F. le Noble , N. Rajewsky , Nature 2013, 495, 333. 23446348 10.1038/nature11928

[18] Q. Yang , F. Li , A. T. He , B. B. Yang , Mol. Ther. 2021, 29, 1683. 33484969 10.1016/j.ymthe.2021.01.018 PMC8116570

[19] Z. Wang , Y. Li , J. Yang , Y. Liang , X. Wang , N. Zhang , X. Kong , B. Chen , L. Wang , W. Zhao , Q. Yang , Cell Death Dis. 2022, 13, 776. 36075896 10.1038/s41419-022-05216-7 PMC9458743

[20] X. Wang , T. Chen , C. Li , W. Li , X. Zhou , Y. Li , D. Luo , N. Zhang , B. Chen , L. Wang , W. Zhao , S. Fu , Q. Yang , J. Hematol. Oncol. 2022, 15, 122. 36038948 10.1186/s13045-022-01345-w PMC9425971

[21] M. Escoll , R. Gargini , A. Cuadrado , I. M. Anton , F. Wandosell , Oncogene 2017, 36. 10.1038/onc.2016.518 28166194

[22] W. Xu , Q. Jin , X. Li , D. Li , X. Fu , N. Chen , Q. Lv , Y. Shi , S. He , L.u Dong , Y. Yang , Y. Yan , F. Shi , Cell Death Dis. 2024, 15, 115. 38326336 10.1038/s41419-024-06505-z PMC10850491

[23] H. Hu , H. Gehart , B. Artegiani , C. LÖpez‐Iglesias , F. Dekkers , O. Basak , J. van Es , S. M. Chuva de Sousa Lopes , H. Begthel , J. Korving , M. van den Born , C. Zou , C. Quirk , L. Chiriboga , C. M. Rice , S. Ma , A. Rios , P. J. Peters , Y. P. de Jong , H. Clevers , Cell 2018, 175, 1591. 30500538 10.1016/j.cell.2018.11.013

[24] I. Tuszynska , M. Magnus , K. Jonak , W. Dawson , J. M. Bujnicki , Nucleic Acids Res. 2015, 43, W425. 25977296 10.1093/nar/gkv493 PMC4489298

[25] S. Shen , Y. Yang , P. Shen , J. Ma , B. Fang , Q. Wang , K. Wang , P. Shi , S. Fan , X. Fang , Ann. Rheum. Dis. 2021, 80, 1209. 34039624 10.1136/annrheumdis-2021-219969 PMC8372377

[26] T. J. Phesse , K. B. Myant , A. M. Cole , R. A. Ridgway , H. Pearson , V. Muncan , G. R. van den Brink , K. H. Vousden , R. Sears , L. T. Vassilev , A. R. Clarke , O. J. Sansom , Cell Death Differ. 2014, 21, 956. 24583641 10.1038/cdd.2014.15 PMC4013513

[27] C. A. Hassig , J. K. Tong , T. C. Fleischer , T. Owa , P. G. Grable , D. E. Ayer , S. L. Schreiber , Proc Natl Acad Sci U S A 1998, 95, 3519. 9520398 10.1073/pnas.95.7.3519 PMC19868

[28] O. Fumiaki , S. Yasushi , I. Satoshi , K. Jun , T. Keiji , Mol. Cell 2016, 64.

[29] P. Liao , S. X. Zeng , X. Zhou , T. Chen , F. Zhou , B. Cao , J. H. Jung , G. S. Del , S. Luo , H. Lu , Mol. Cell 2017, 68. 10.1016/j.molcel.2017.11.006 PMC6204219 29225033

[30] L. Yi , Z. Meng‐Chen , X. Xiao‐Kang , Z. Yang , M. Chatoo , Z. Tao , D. Hong , N. Eviatar , D. Ji‐Zeng , C. Xue‐Qun , Front Endocrinol (Lausanne) 2019, 10, 152.30915036

[31] G. Bianchini , J. M. Balko , I. A. Mayer , M. E. Sanders , L. Gianni , Nat. Rev. Clin. Oncol. 2016, 13, 674. 27184417 10.1038/nrclinonc.2016.66 PMC5461122

[32] Y.‐Z. Jiang , D. Ma , C. Suo , J. Shi , M. Xue , X. Hu , Y. Xiao , K.‐D. Yu , Y.‐R. Liu , Y. Yu , Y. Zheng , X. Li , C. Zhang , P. Hu , J. Zhang , Q. Hua , J. Zhang , W. Hou , L. Ren , D. Bao , B. Li , J. Yang , L. Yao , W. J. Zuo , S. Zhao , Y. Gong , Y.‐X. Ren , Y.‐X. Zhao , Y. S. Yang , Z. Niu , et al., Cancer Cell 2019, 35, 428. 30853353 10.1016/j.ccell.2019.02.001

[33] D. Dibra , S. M. Moyer , A. K. EI‐Naggar , Y. Qi , X. Su , G. Lozano , Proc. Natl. Acad. Sci. USA 2023, 120, e2308807120. 37579145 10.1073/pnas.2308807120 PMC10450424

[34] J. Liu , C. Zhang , D. Xu , T. Zhang , C. Chang , J. Wang , J. Liu , L. Zhang , B. G. Haffty , W. Zong , W. Hu , Z. Feng , J. Clin. Invest. 2023, 133. 10.1172/JCI164354 PMC10014102 36749630

[35] F. Liang , Q. Luo , H. Han , J. Zhang , Y. Yang , J. Chen , Acta Biochim Biophys Sin (Shanghai) 2023, 55, 367. 36942988 10.3724/abbs.2023021 PMC10160232

[36] Y. Zhao , Y. Li , J. Sheng , F. Wu , K. Li , R. Huang , X. Wang , T. Jiao , X. Guan , Y. Lu , X. Chen , Z. Luo , Y. Zhou , H. Hu , W. Liu , B. Du , S. Miao , J. Cai , L. Wang , H. Zhao , J. Ying , X. Bi , J. Exp. Clin. Cancer Res. 2019, 38. 10.1186/s13046-019-1375-9 PMC6712617 31455383

[37] R. Takahashi , F. Takeshita , K. Honma , M. Ono , K. Kato , T. Ochiya , Sci. Rep. 2013, 3. 10.1038/srep02474 PMC3747512 23959174

[38] H. Solomon , N. Dinowitz , I. S. Pateras , T. Cooks , Y. Shetzer , A. Molchadsky , M. Charni , S. Rabani , G. Koifman , O. Tarcic , Z. Porat , I. Kogan‐Sakin , N. Goldfinger , M. Oren , C. C. Harris , V. G. Gorgoulis , V. Rotter , Oncogene 2018, 37, 1669. 29343849 10.1038/s41388-017-0060-8 PMC6448595

[39] M. Escoll , R. Gargini , A. Cuadrado , I. M. Anton , F. Wandosell , Oncogene 2017, 36, 3515. 28166194 10.1038/onc.2016.518

[40] G. van Niekerk , L. M. Davids , S. M. Hattingh , A. M. Engelbrecht , Int. J. Cancer 2017, 140, 993. 27676693 10.1002/ijc.30448

[41] A. W. Lambert , R. A. Weinberg , Nat. Rev. Cancer 2021, 21, 325. 33547455 10.1038/s41568-021-00332-6

[42] L. Liu , T. Tao , S. Liu , X. Yang , X. Chen , J. Liang , R. Hong , W. Wang , Y.i Yang , X. Li , Y. Zhang , Q. Li , S. Liang , H. Yu , Y. Wu , X. Guo , Y. Lai , X. Ding , H. Guan , J. Wu , X. Zhu , J. Yuan , J. Li , S. Su , M. Li , X. Cai , J. Cai , H. Tian , Nat. Commun. 2021, 12, 2693. 33976158 10.1038/s41467-021-22971-x PMC8113560

[43] J. P. Sullivan , J. D. Minna , J. W. Shay , Cancer Metastasis Rev. 2010, 29, 61. 20094757 10.1007/s10555-010-9216-5 PMC2864581

[44] X. Bai , J. Ni , J. Beretov , S. Wang , X. Dong , P. Graham , Y. Li , Adv. Sci. (Weinh) 2021, 8, e2102658. 34708581 10.1002/advs.202102658 PMC8693071

[45] N. C. D'Amato , J. H. Ostrander , M. L. Bowie , C. Sistrunk , A. Borowsky , R. D. Cardiff , K. Bell , L. J. T. Young , K. Simin , R. E. Bachelder , J. Delrow , A. Dawson , L. D. Yee , K. Mrózek , T. M. Clay , T. Osada , V. L. Seewaldt , PLoS One 2012, 7, e45684. 23049838 10.1371/journal.pone.0045684 PMC3458110

[46] J. E. Audia , R. M. Campbell , Cold Spring Harb Perspect Biol. 2016, 8. 10.1101/cshperspect.a019521 PMC4817802 27037415

[47] L. S. Dunaway , J. S. Pollock , Cardiovasc. Res. 2022, 118, 1885. 34264338 10.1093/cvr/cvab198 PMC9239577

[48] L. Zhang , L. Bu , J. Hu , Z. Xu , L. Ruan , Y. Fang , P. Wang , Biol. Chem. 2018, 399, 603. 29537214 10.1515/hsz-2017-0306

[49] P. Ma , R. M. Schultz , Cell Death Differ. 2016, 23. 10.1038/cdd.2016.31 PMC4946893 27082454

[50] S. K. Madden , A. D. de Araujo , M. Gerhardt , D. P. Fairlie , J. M. Mason , Mol Cancer 2021, 20, 3. 33397405 10.1186/s12943-020-01291-6 PMC7780693

[51] M. J. Duffy , S. O'Grady , M. Tang , J. Crown , Cancer Treat. Rev. 2021, 94, 102154. 33524794 10.1016/j.ctrv.2021.102154

[52] F. Y. Gao , X. T. Li , K. Xu , R. T. Wang , X. X. Guan , Cell Commun. Signal 2023, 21, 28. 36721232 10.1186/s12964-023-01043-1 PMC9887805

[53] Z. Y. Li , Y. Xie , M. Deng , L. Zhu , X. Wu , G. Li , N.‐X.i Shi , C. Wen , W. Huang , Y. Duan , Z. Yin , X. J. Lin , Cancer Lett. 2022, 526, 322. 34767926 10.1016/j.canlet.2021.11.006

[54] D. Q. Calcagno , M. F. Leal , P. P. Assumpção , M. A. C. Smith , R. R. Burbano , World J. Gastroenterol. 2008, 14, 5962. 18932273 10.3748/wjg.14.5962 PMC2760197

[55] M. L. Jin , K. W. Jeong , Exp. Mol. Med. 2023, 55, 1333. 37394580 10.1038/s12276-023-01014-z PMC10394043

[56] J.‐W. Jiao , X. H. Zhan , J. J. Wang , L.‐X. He , Z. C. Guo , X. E. Xu , L.‐D. Liao , X. Huang , B. Wen , Y.‐W. Xu , H. Hu , G. Neufeld , Z. J. Chang , K. Zhang , L.‐Y. Xu , E.‐M. Li , Redox Biol. 2022, 57, 102496. 36209516 10.1016/j.redox.2022.102496 PMC9547286

[57] S. G. Swygert , S. Senapati , M. F. Bolukbasi , S. A. Wolfe , S. Lindsay , C. L. Peterson , Proc. Natl. Acad. Sci. USA 2018, 115, 12447. 30455303 10.1073/pnas.1810647115 PMC6298083

[58] X. Huo , J. Zhang , J. Cell. Mol. Med. 2005, 9, 103. 15784168 10.1111/j.1582-4934.2005.tb00340.x PMC6741356

[59] G. R. Vanaja , H. G. Ramulu , A. M. Kalle , Cell Commun Signal 2018, 16, 20. 29716651 10.1186/s12964-018-0231-4 PMC5930436

[60] Y. Zhang , X. Long , X. Ruan , Q. Wei , L. Zhang , L. Wo , D. Huang , L. Lin , D. Wang , L.i Xia , Q. Zhao , J. Liu , Q. Zhao , M. He , Cell Discov. 2021, 7, 93. 34642310 10.1038/s41421-021-00326-6 PMC8511299

[61] Z. Zhang , X. Fang , X. Wu , L. Ling , F. Chu , J. Li , S. Wang , J. Zang , B. Zhang , S. Ye , L. Zhang , B. Yang , S. Lin , H. Huang , A. Wang , F. Zhou , Mol. Cell 2020, 79, 304. 32679077 10.1016/j.molcel.2020.06.020

[62] N. Stojanovic , Z. Hassan , M. Wirth , P. Wenzel , M. Beyer , C. Schäfer , P. Brand , A. Kroemer , R. H. Stauber , R. M. Schmid , A. Arlt , A. Sellmer , S. Mahboobi , R. Rad , M. Reichert , D. Saur , O. H. Krämer , G. Schneider , Oncogene 2017, 36, 1804. 27721407 10.1038/onc.2016.344

[63] B. Roy , J. Beamon , E. Balint , B. Reisman , Mol. Cell. Biol. 1994, 14, 7805. 7969121 10.1128/mcb.14.12.7805-7815.1994 PMC359320

[64] P. Liao , S. X. Zeng , X. Zhou , T. Chen , F. Zhou , B.o Cao , J. H. Jung , G. Del Sal , S. Luo , H. Lu , Mol. Cell 2017, 68, 1134. 29225033 10.1016/j.molcel.2017.11.006 PMC6204219

[65] I. López‐Sánchez , A. Valbuena , M. Vázquez‐Cedeira , J. Khadake , M. Sanz‐García , A. Carrillo‐Jiménez , P. A. Lazo , FEBS Lett. 2014, 588, 692. 24492002 10.1016/j.febslet.2014.01.040

[66] R. Magrini , D. Russo , G. Fronza , A. Inga , P. Menichini , J. Cell. Biochem. 2007, 100, 1276. 17063487 10.1002/jcb.21122

[67] A. Akinleye , Z. Rasool , J. Hematol. Oncol. 2019, 12, 92. 31488176 10.1186/s13045-019-0779-5 PMC6729004

[68] E. B. Garon , N. A. Rizvi , R. Hui , N. Leighl , A. S. Balmanoukian , J. P. Eder , A. Patnaik , C. Aggarwal , M. Gubens , L. Horn , E. Carcereny , M.‐J.u Ahn , E. Felip , J. S. Lee , M. D. Hellmann , O. Hamid , J. W. Goldman , J.‐C. Soria , M. Dolled‐Filhart , R. Z. Rutledge , J. Zhang , J. K. Lunceford , R. Rangwala , G. M. Lubiniecki , C. Roach , K. Emancipator , L. Gandhi , N. Engl. J. Med. 2015, 372, 2018. 25891174 10.1056/NEJMoa1501824

[69] L. Stanek , et al., Bratisl Lek Listy 2022, 123, 83. 35065582 10.4149/BLL_2022_013

[70] I. Pérez‐Nunez , C. Rozalén , J. Á. Palomeque , I. Sangrador , M. Dalmau , L. Comerma , A. Hernández‐Prat , D. Casadevall , S. Menendez , D. D. Liu , M. Shen , J. Berenguer , I. R. Ruiz , R. Peña , J. C. Montañés , M. M. Albà , S. Bonnin , J. Ponomarenko , R. R. Gomis , J. M. Cejalvo , S. Servitja , D. M. Marzese , L. Morey , L. Voorwerk , J. Arribas , B. Bermejo , M. Kok , L. Pusztai , Y. Kang , J. Albanell , et al., Nat. Cancer 2022, 3, 355. 35301507 10.1038/s43018-022-00339-4

[71] M. A. Cortez , C. Ivan , D. Valdecanas , X. Wang , H. J. Peltier , Y. Ye , L. Araujo , D. P. Carbone , K. Shilo , D. K. Giri , K. Kelnar , D. Martin , R. Komaki , D. R. Gomez , S. Krishnan , G. A. Calin , A. G. Bader , J. W. Welsh , J. Natl. Cancer Inst. 2016, 108. 10.1093/jnci/djv303 PMC4862407 26577528

[72] N. Liu , X. Jiang , L. Guo , C. Zhang , M. Jiang , Z. Sun , Y. Zhang , W. Mi , J. Li , Y. Fu , F. Wang , L. Zhang , Y. Zhang , Int. J. Biol. Sci. 2022, 18, 2419. 35414774 10.7150/ijbs.67200 PMC8990467

[73] T. J. Curiel , S. Wei , H. Dong , X. Alvarez , P. Cheng , P. Mottram , R. Krzysiek , K. L. Knutson , B. Daniel , M. C. Zimmermann , O. David , M. Burow , A. Gordon , N. Dhurandhar , L. Myers , R. Berggren , A. Hemminki , R. D. Alvarez , D. Emilie , D. T. Curiel , L. Chen , W. Zou , Nat. Med. 2003, 9, 562. 12704383 10.1038/nm863

[74] T. Alexander , et al., J. Exp. Clin. Cancer Res. 2019, 38.

[75] D. Zimmerli , C. S. Brambillasca , F. Talens , J. Bhin , R. Linstra , L. Romanens , A. Bhattacharya , S. E. P. Joosten , A. M. Da Silva , N. Padrao , M. D. Wellenstein , K. Kersten , M. de Boo , M. Roorda , L. Henneman , R. de Bruijn , S. Annunziato , E. van der Burg , A. P. Drenth , C. Lutz , T. Endres , M. van de Ven , M. Eilers , L. Wessels , K. E. de Visser , W. Zwart , R. S. N. Fehrmann , M. A. T. M. van Vugt , J. Jonkers , Nat. Commun. 2022, 13, 6579. 36323660 10.1038/s41467-022-34000-6 PMC9630413

[76] W. Jing , G. Wang , Z. Cui , G. Xiong , X. Jiang , Y. Li , W. Li , B.o Han , S. Chen , B. Shi , Cancer Res. 2022, 82, 114. 34753771 10.1158/0008-5472.CAN-21-2362

