# Supplementary material for: CircCFL1 Promotes TNBC Stemness and Immunoescape via Deacetylation‐Mediated c‐Myc Deubiquitylation to Facilitate Mutant TP53 Transcription
Source: Adv Sci (Weinh). 2024 Jul 9;11(34):2404628. doi: 10.1002/advs.202404628 (PMC11425638; doi:10.1002/advs.202404628)
Supplement: Supplementary file 1 — Supporting Information [file ADVS-11-2404628-s001.docx]

Supplementary Figures and Tables for

**CircCFL1 promotes TNBC stemness and immunoescape via deacetylation-mediated c-Myc deubiquitylation to facilitate mutant TP53 transcription**

Zekun Wang^1#^, Yaming Li^1#^, Jingwen Yang^1^, Yuhan Sun^1^, Yinqiao He^1^, Yuping Wang^2^, Yiran Liang^1^, Xi Chen^1^, Tong Chen^1^, Dianwen Han^1^, Ning Zhang^1^, Bing Chen^3^, Wenjing Zhao^3^, Lijuan Wang^3^, Dan Luo^3^ and Qifeng Yang^1,3,4*^

^*^**Address correspondence to:** Qifeng Yang (qifengy_sdu@163.com).

**Supplementary Figures**

**
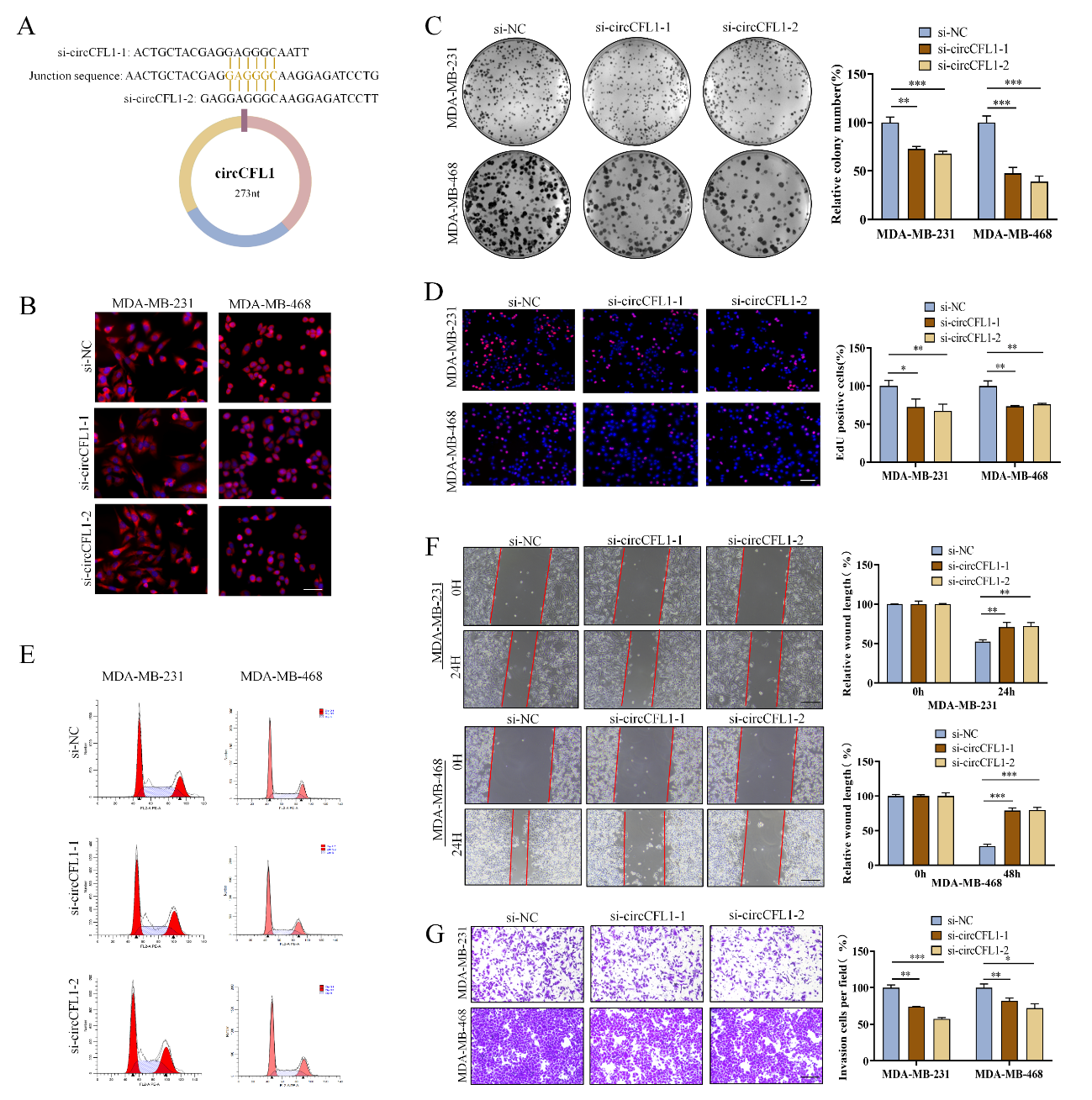
**

**Figure S1.** A. Schematic illustration showing the sequences of the siRNAs specific for the backsplicing junction of circCFL1. B. FISH assays were performed to detect the efficiency of circCFL1 interference. Scale bars=50 μm. C. Colony formation assays were used to determine the proliferation rate of TNBC cells after transfection with si-circCFL1. EdU (D) and flow cytometry (E) assays were performed to evaluate the proliferation of TNBC cells with circCFL1 knockdown. Scale bars=200 μm. Wound healing (F) and Transwell (G) assays illustrated the effects of circCFL1 knockdown on the migration and invasion abilities of TNBC cells. Scale bars=200 μm. *p<0.05; **p<0.01; ***p<0.001.


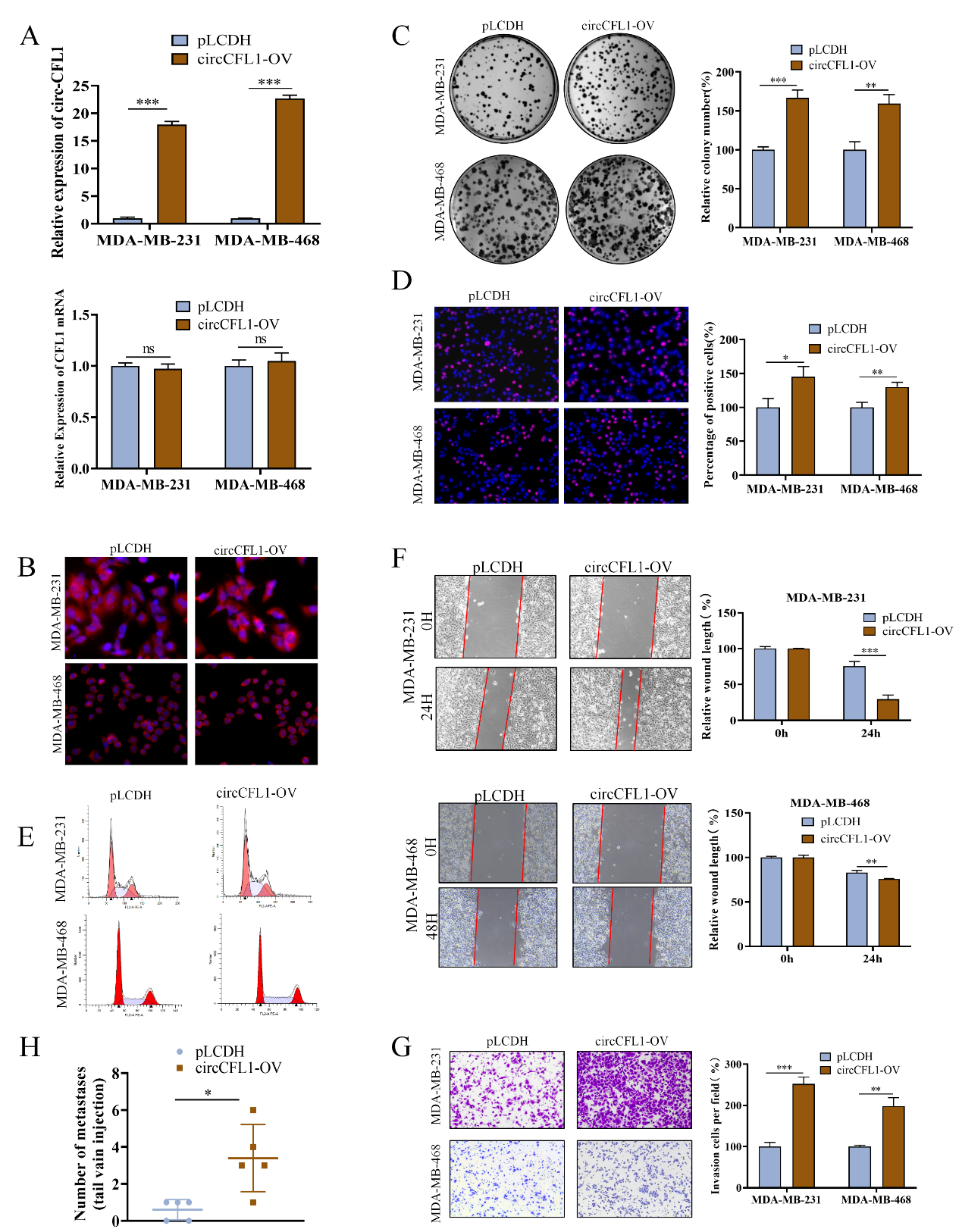


**Figure S2. A.** The overexpression efficiency of circCFL1 and its effect on CFL1 mRNA were measured using qRT‒PCR assays. **B.** FISH assays were performed to detect the efficiency of circCFL1 overexpression. Scale **bars=50 μm.** Colony formation assays were used to determine the proliferation rate of TNBC cells after transfection with circCFL1 overexpression vectors. EdU (**D**) and flow cytometry (**E**) assays were performed to detect the proliferation of TNBC cells overexpressing circCFL1. Scale bars=200 μm. Wound healing (**F**) and Transwell (**G**) assays illustrated the effects of cirCFL1 overexpression on the migration and invasion rates of TNBC cells. Scale bars=200 μm. H. Number of metastatic lung nodules in BALB/c nude mice (n = 5 mice in each group). ns nonsignificant; *p<0.05; **p<0.01; ***p<0.001.


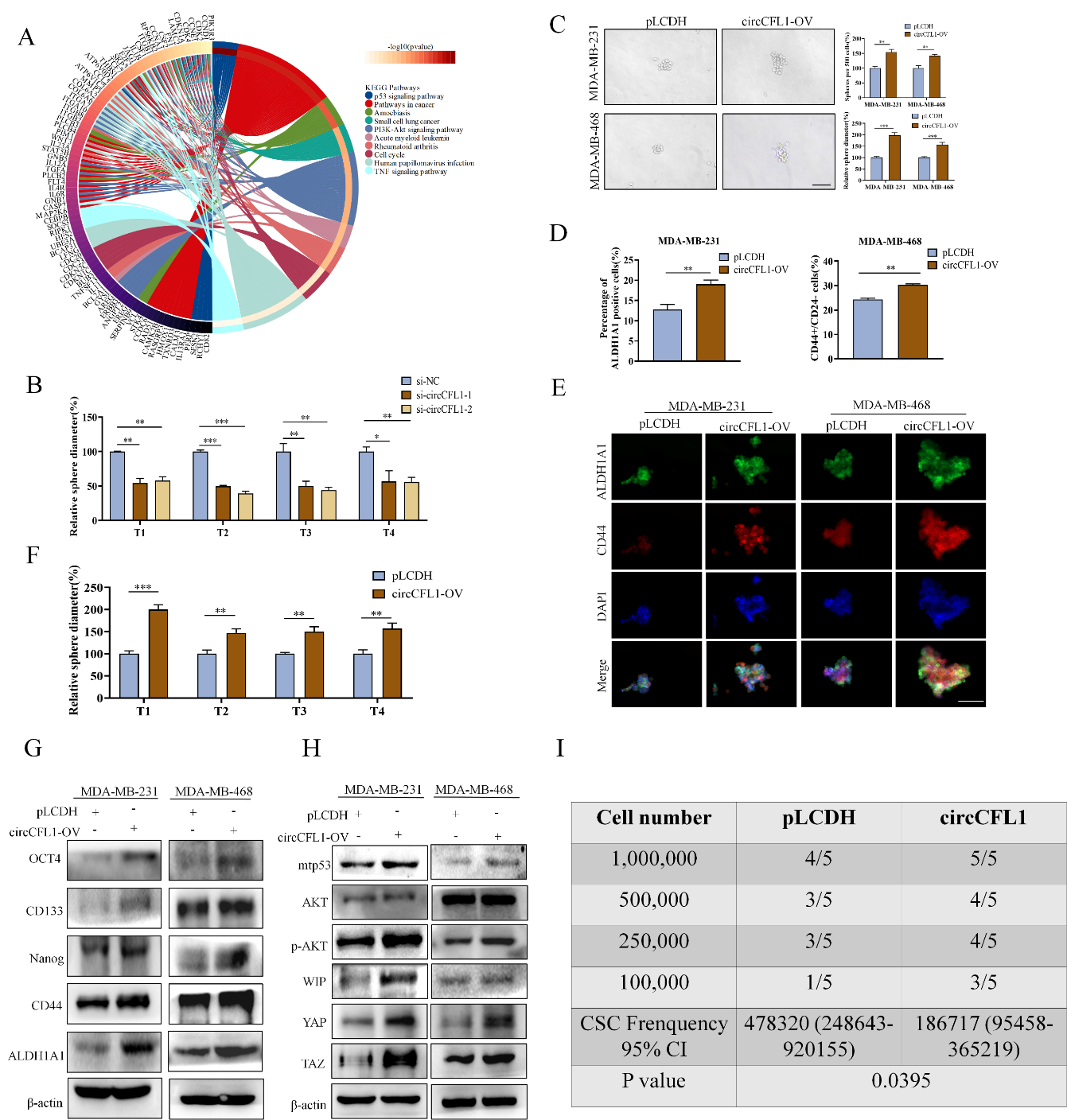


**Figure S3. A.** KEGG enrichment analysis of differentially expressed genes in circCFL1-knockdown MDA-MB-231 cells based on RNA-seq data. **B**. Statistical analysis of the growth rate of PDOs in which circCFL1 was silenced. **C.** Sphere formation assays were performed to assess the stemness of MDA-MB-231 and MDA-MB-468 cells after transfection with the circCFL1 overexpression vector. Scale bars=100 μm. The statistical analysis of the flow cytometry data illustrated the difference in stemness after circCFL1 overexpression. **E.** IF assays were performed to examine the expression of ALDH1A1 and CD44 in TNBC cells overexpressing circCFL1. Scale bars=100 μm. **F.** Statistical analysis of the growth rate of PDOs overexpressing circCFL1. **G.** Western blotting assays illustrated that the expression of stemness-associated proteins was altered after circCFL1 overexpression. **H.** Overexpression of circCFL1 promoted the expression of mutp53 and further promoted the p-AKT/WIP/YAP/TAZ signaling pathway. **I.** Statistical table of limiting dilution assays in vivo. *p<0.05; **p<0.01; ***p<0.001.


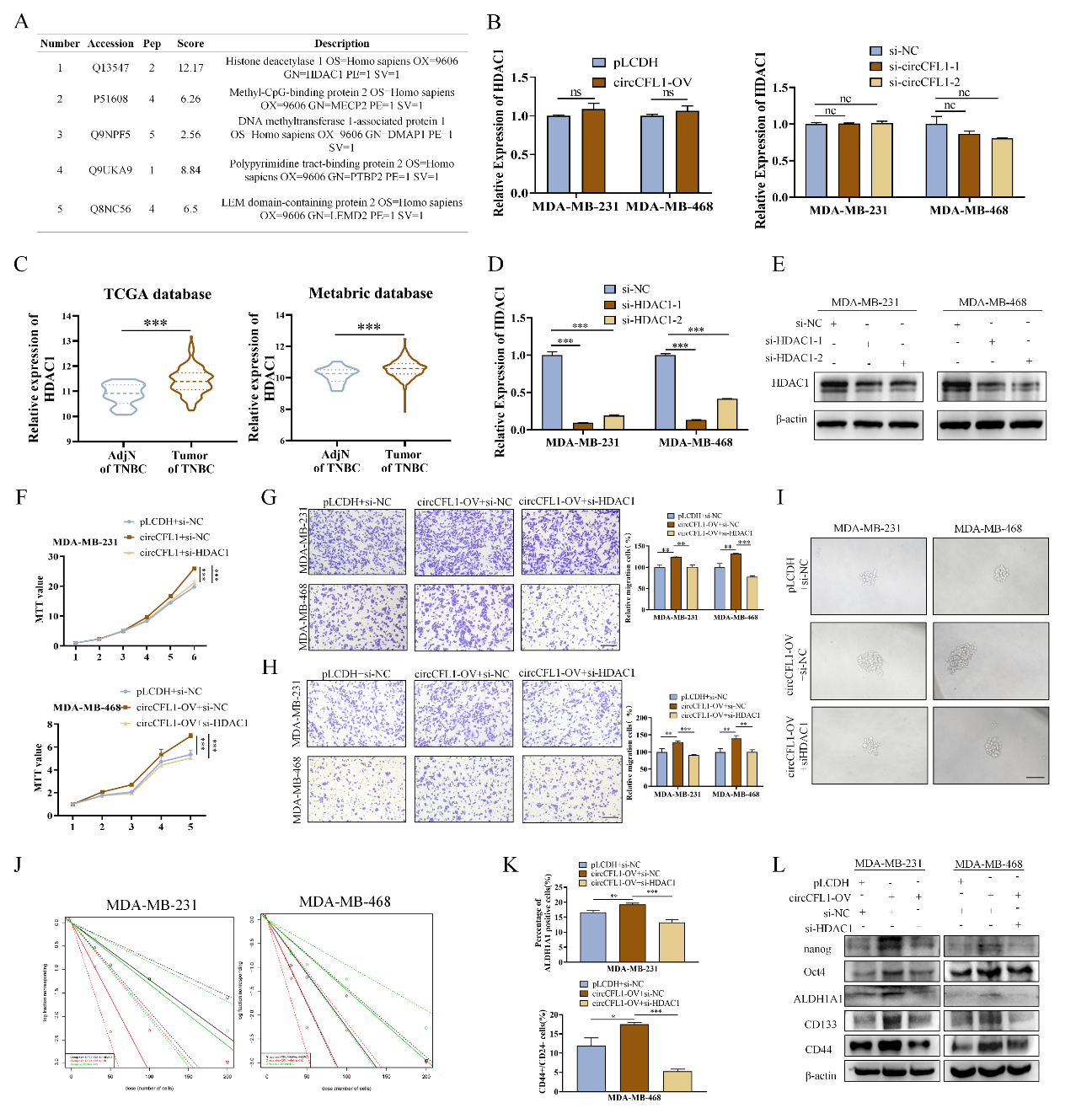


**Figure S4. A.** The top five proteins pulled down by circCFL1. **B.** The impacts of circCFL1 overexpression or interference on HDAC1 at the RNA level. **C.** Analyses of TCGA and METABRIC data illustrated the expression of HDAC1 in adjacent normal TNBC tissues and TNBC tissues. **D, E.** Interference efficiency of HDAC1. **F.** MTT assays were performed to detect the proliferation of TNBC cells after circCFL1 overexpression with or without HDAC1 interference. **G-H.** Transwell assays were used to verify the migration and invasion abilities of cells after circCFL1 overexpression or HDAC1 inhibition. Scale bars=200 μm. Sphere formation (**I**), limiting dilution (**J**), and flow cytometry (**K**) assays were performed to detect the stemness of TNBC cells after circCFL1 overexpression with or without HDAC1 knockdown. Scale bars=100 μm. **L.** Western blotting assays showing the expression levels of stemness-associated proteins after circCFL1 overexpression with or without HDAC1 knockdown. *p<0.05; **p<0.01; ***p<0.001.


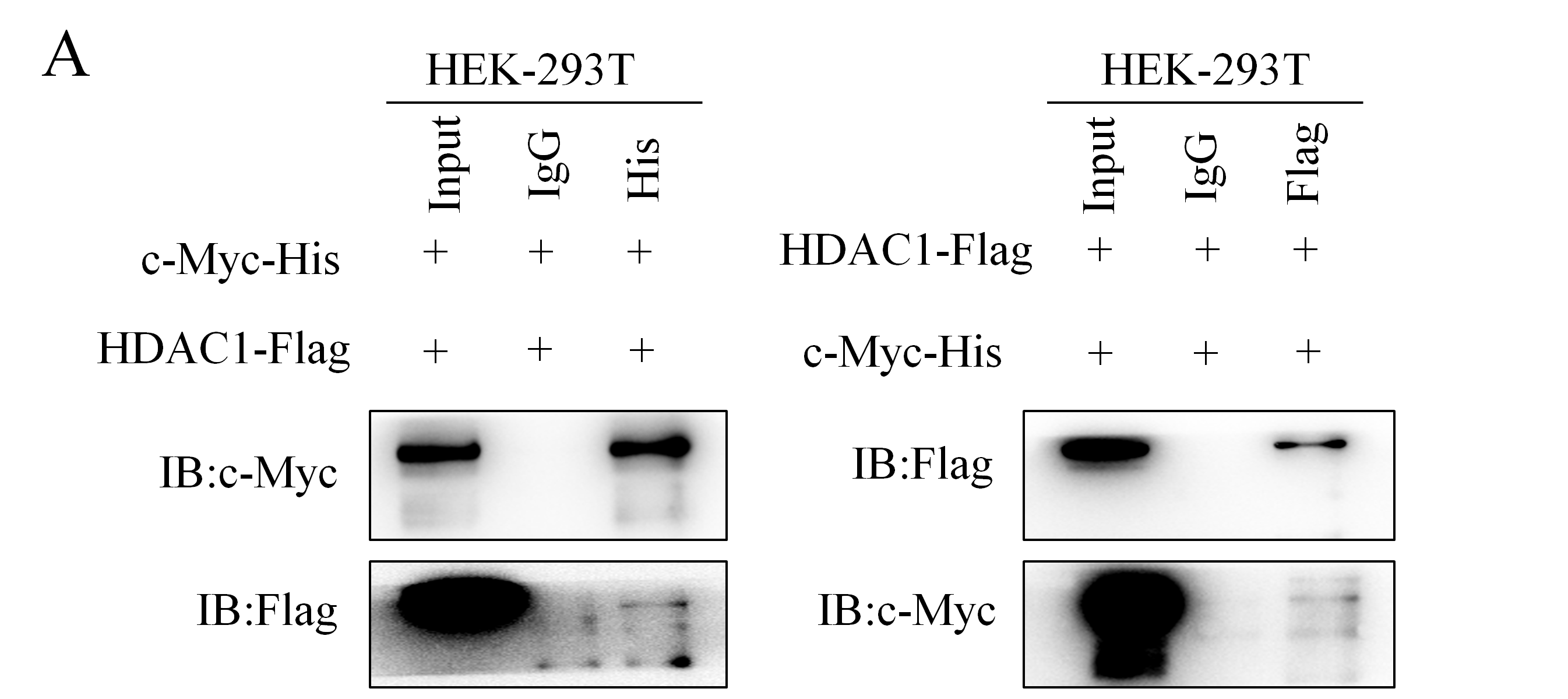


**Figure S5. A.** Coimmunoprecipitation assays were performed in HEK-293T cells to identify the interaction between c-Myc and HDAC1.


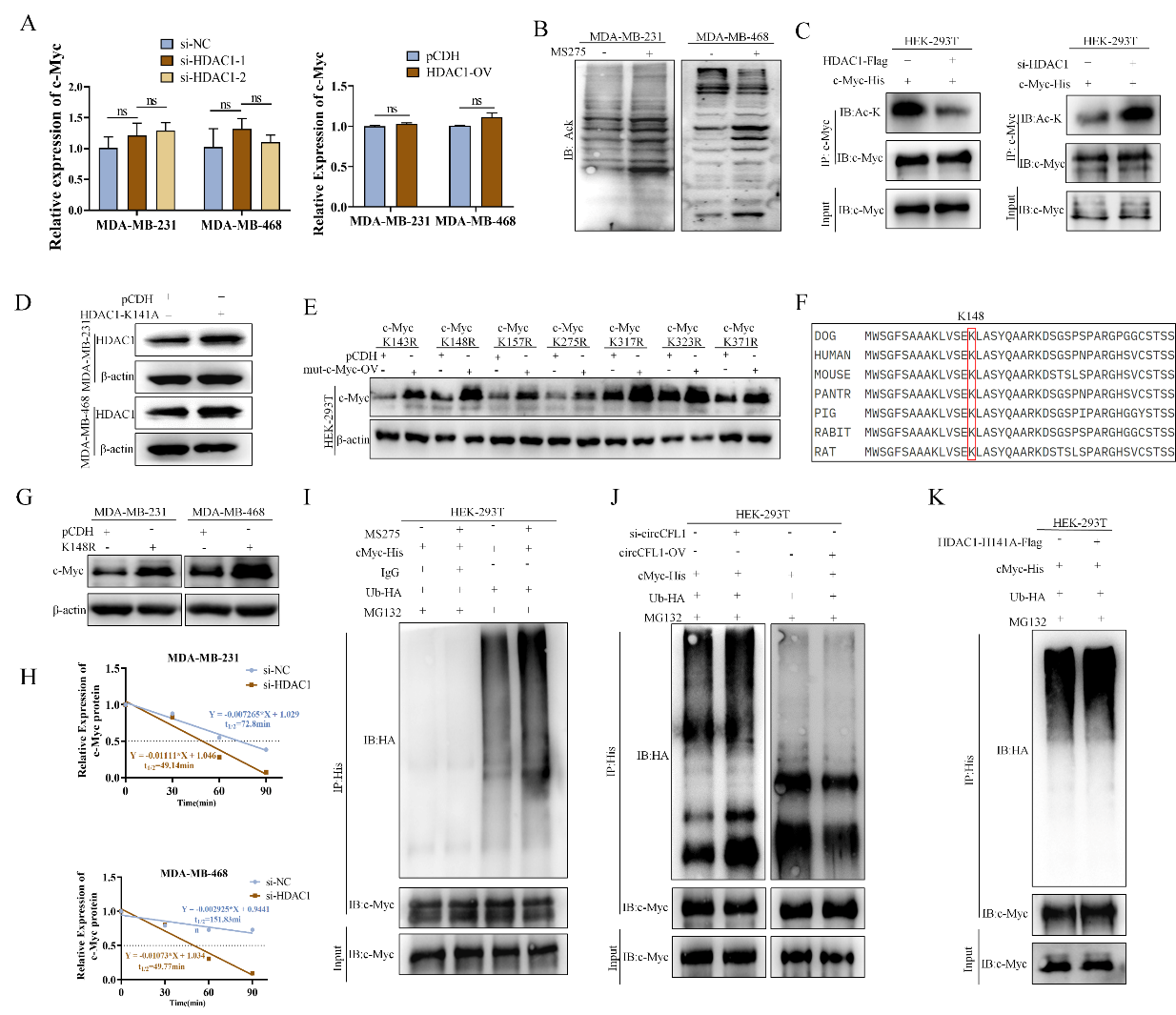


**Figure S6. A.** qRT‒PCR assays showing the mRNA levels of c-Myc after HDAC1 interference or overexpression. **B.** Western blotting assays were performed to determine the acetylation levels of the TMNC cell lines after treatment with MS275. **C.** Acetylation levels of c-Myc after overexpression or knockdown of HDAC1. **D.** Western blotting assays confirmed the transfection efficiency of HDAC1-K141A in TNBC cells. **E.** Transfection efficiencies of the 7 mutant isoforms of c-Myc were verified in HEK-293T cells. **F.** Conservation of the c-Myc K148 site across different species. **G.** Western blotting assays were used to determine the transfection efficiency of c-Myc-K148R in TNBC cells. **H.** ImageJ software was used to quantify the band intensity of the western blot after treatment with CHX for the indicated times. **I.** Western blotting was performed to evaluate the levels of ubiquitinated c-Myc after treatment with MS275. **J.** Western blotting was performed to evaluate changes in c-Myc ubiquitination levels in cells with circCFL1 knockdown or overexpression. **K.** Western blotting assay showing the levels of ubiquitinated c-Myc after transfection with the HDAC1-H141A vector. ns nonsignificant.


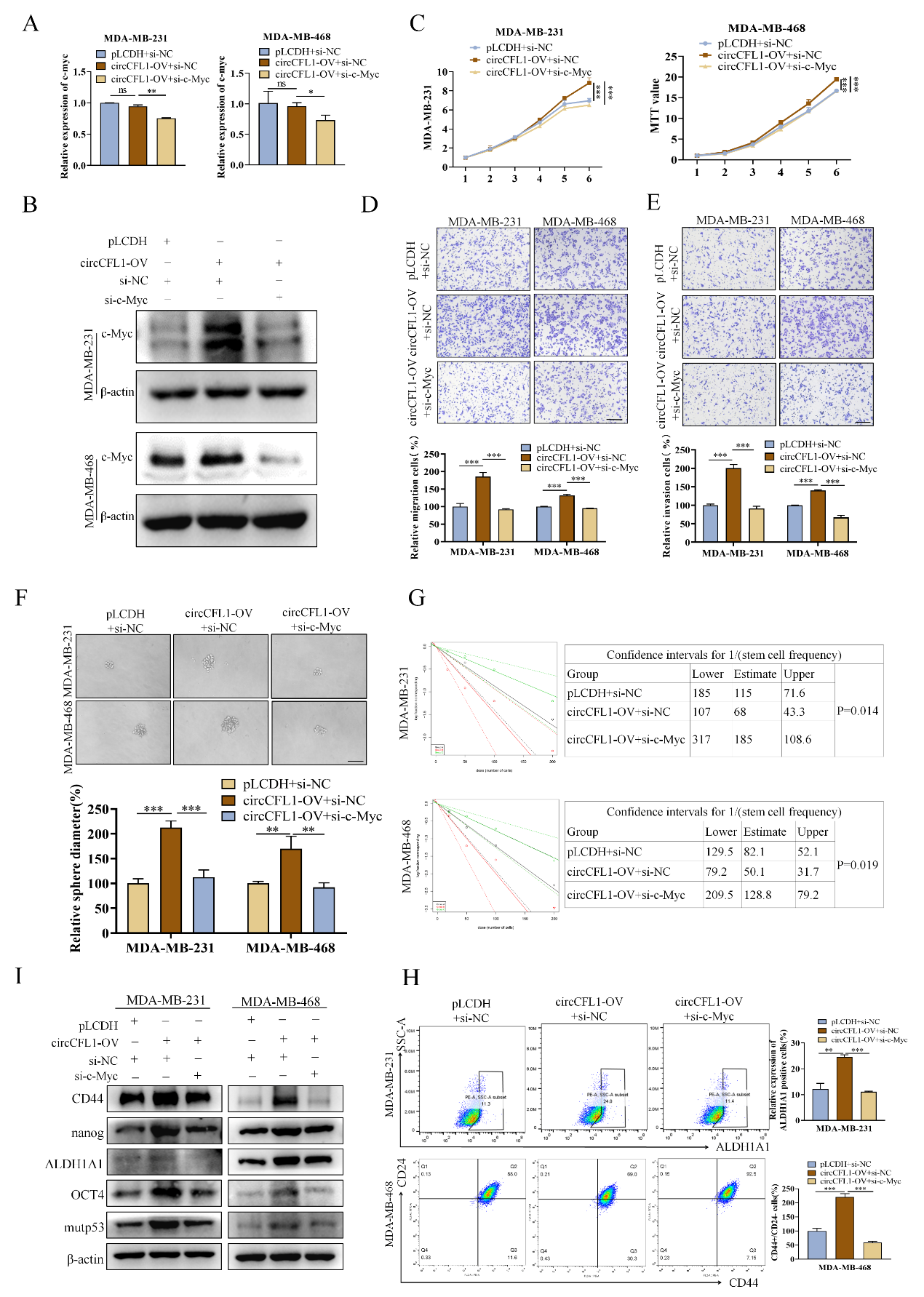


**Figure S7.** qRT‒PCR (**A**) and western blotting (**B**) were performed to determine the c-Myc levels in TNBC cells after transfection with the circCFL1 overexpression vector and c-Myc siRNA. **C.** MTT assays showing the proliferation of TNBC cells after transfection with the circCFL1 overexpression vector and c-Myc siRNA. **D-E.** Transwell assays were used to verify the influence of circCFL1 and c-Myc on migration and invasion abilities. Scale bars=200 μm. Sphere formation (**F**), limiting dilution (**G**), and flow cytometry (**H**) assays were performed to detect the stemness of TNBC cells after circCFL1 overexpression and c-Myc knockdown. Scale bars=100 μm. **I.** Western blotting assays showing the expression levels of stemness-associated proteins after circCFL1 overexpression and c-Myc knockdown. ns nonsignificant; *p<0.05; **p<0.01; ***p<0.001.


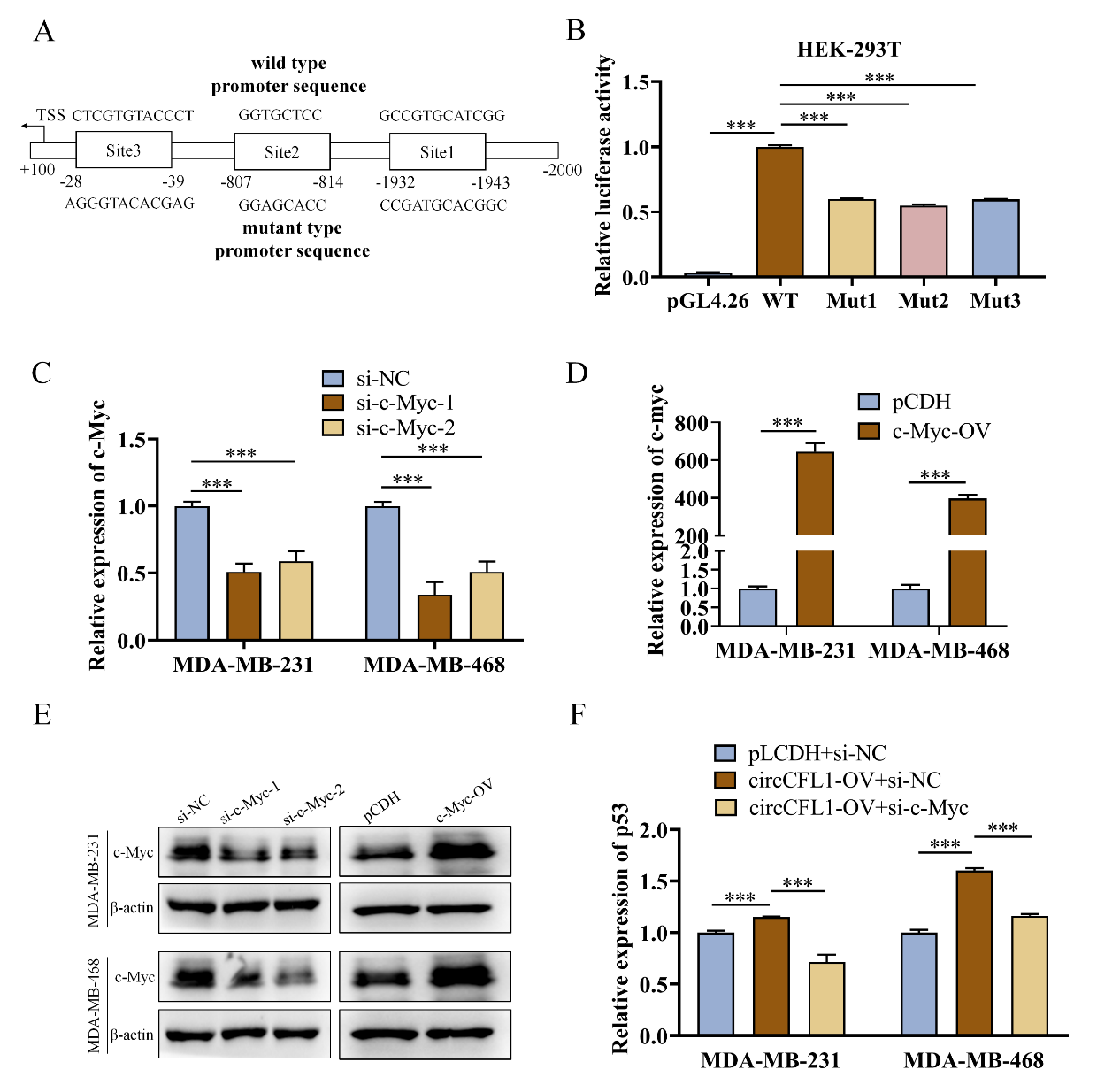


**Figure S8. A.** Schematic illustration showing the mutant sequence of the TP53 promoter used in this study. **B.** Dual-luciferase reporter assays showing the transcriptional activities of TP53 promoters with different mutant sequences. **C-E.** Transfection efficiency of c-Myc knockdown and overexpression in TNBC cells. **F.** Effects of circCFL1 overexpression and c-Myc knockdown on the expression levels of TP53 in MDA-MB-231 and MDA-MB-468 cells. ***p<0.001.


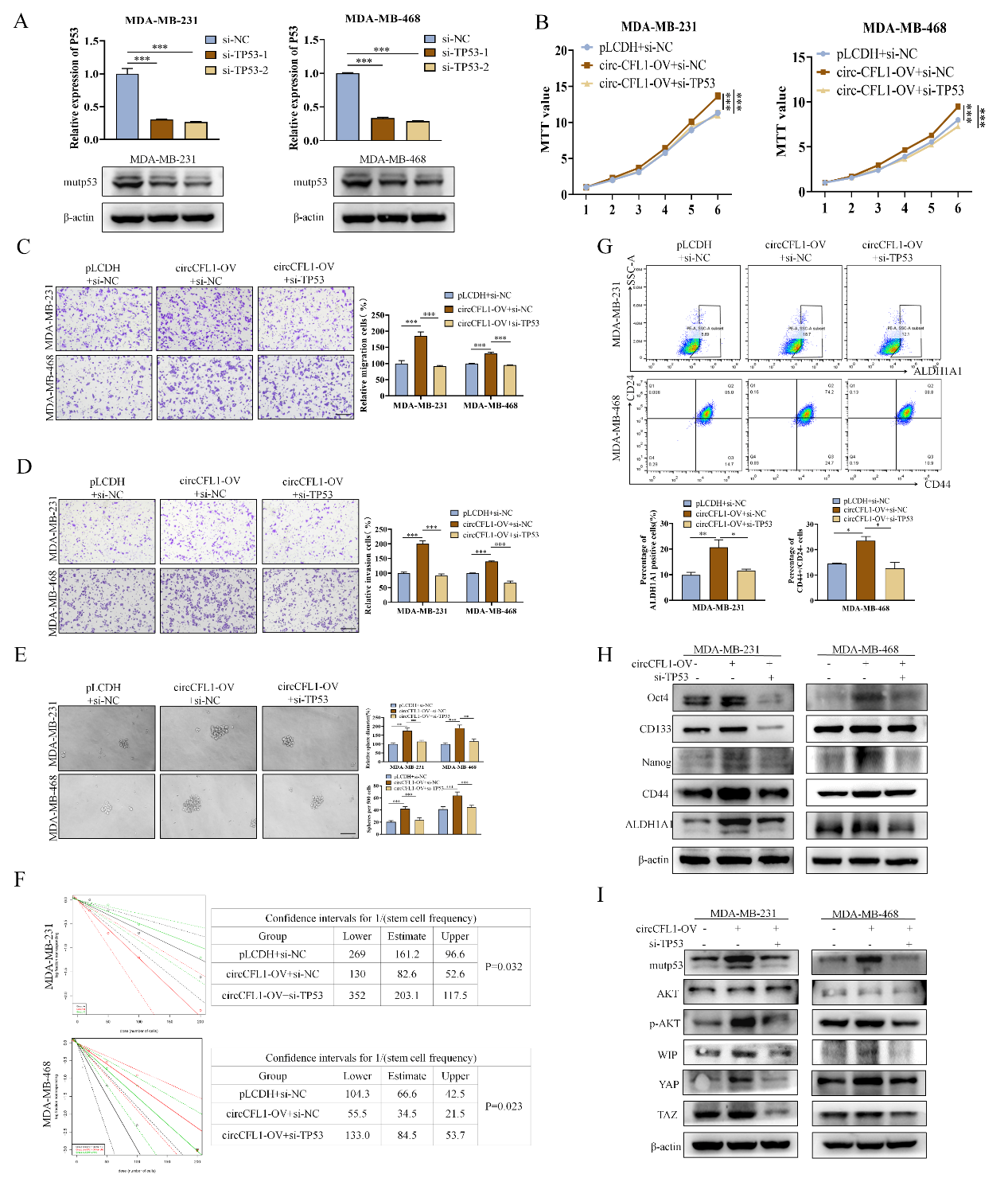


**Figure S9. A.** Transfection efficiencies of TP53 siRNAs. **B.** MTT assays showing the proliferation rate of TNBC cells after transfection with the circCFL1 overexpression vector and TP53 siRNA. **C-D.** Transwell assays were used to verify the migration and invasion abilities of TNBC cells after circCFL1 overexpression or mutp53 knockdown. Scale bars=200 μm. Sphere formation (**E**), limiting dilution (**F**), and flow cytometry (**G**) assays were performed to detect the stemness of TNBC cells after circCFL1 overexpression or mutp53 knockdown. Scale bars=100 μm. **H.** Western blotting assays showing the expression levels of stemness-associated proteins after circCFL1 overexpression or mutp53 knockdown. **I.** The expression levels of mutp53 and the activation of the p-AKT/WIP/YAP/TAZ signaling pathway were examined in TNBC cells with circCFL1 overexpression and mutp53 knockdown. *p<0.05; **p<0.01; ***p<0.001.


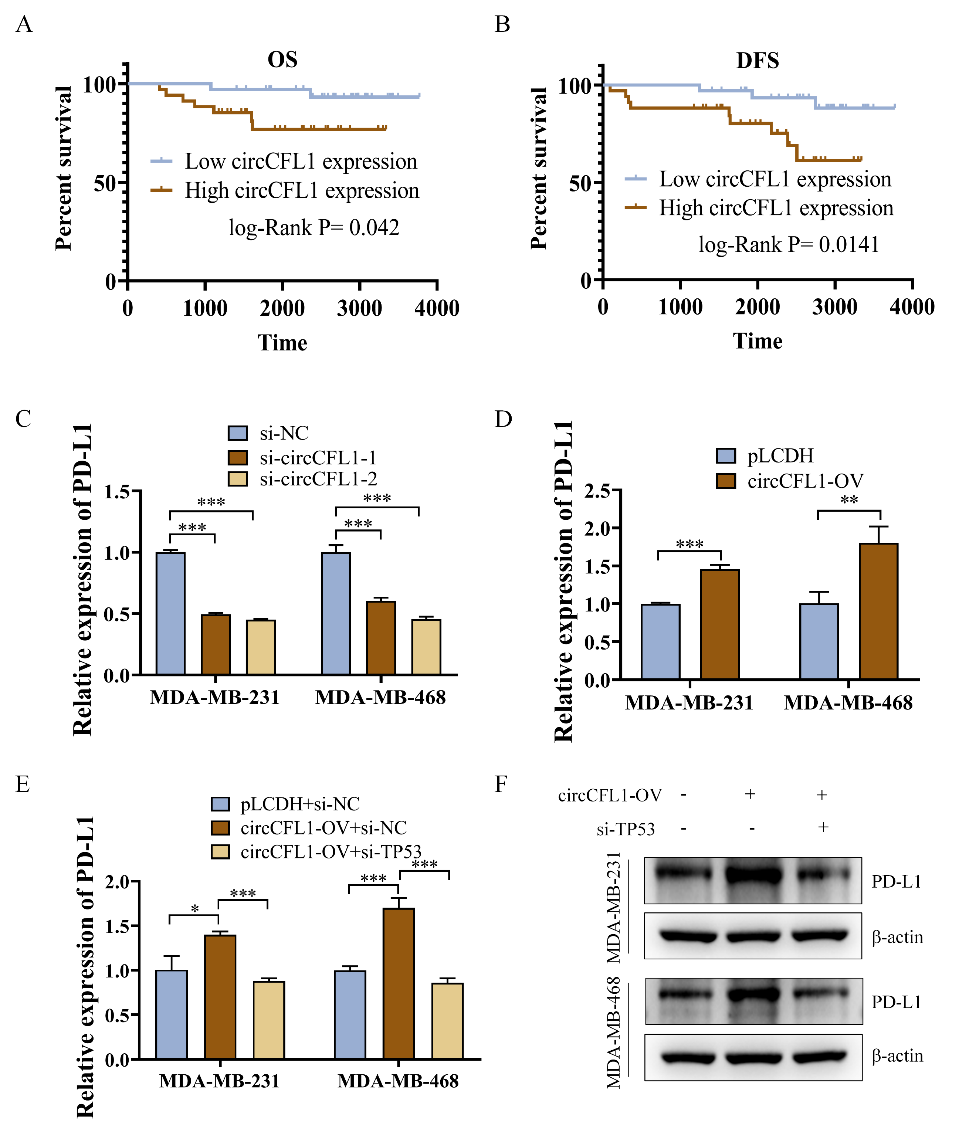


**Figure S10. A-B.** Kaplan–Meier survival analysis of the prognostic value of circCFL1 for both OS and DFS in TNBC patients with mutant TP53. qRT‒PCR assays showing the mRNA levels of PD-L1 after circCFL1 interference (**C**) or overexpression (**D**). **E.** Effects of circCFL1 overexpression and mutant TP53 knockdown on the expression levels of PD-L1 in MDA-MB-231 and MDA-MB-468 cells. **F.** Western blotting assays showing the expression levels of PD-L1 after circCFL1 overexpression and mutant TP53 knockdown. *p<0.05; **p<0.01; ***p<0.001.

**Supplementary Tables**

**Table S1. Antibodies used in the experiments.**

| **Antigen** | **Supplier** | **Catalog #** | **Application** |
| --- | --- | --- | --- |
| CyclinD1 | Cell Signaling  Technology | 55506S | WB: 1:1000 |
| CyclinB1 | Cell Signaling  Technology | 12231 | WB: 1:1000 |
| CDK6 | Cell Signaling  Technology | 13331 | WB: 1:1000 |
| CDK4 | Cell Signaling  Technology | 12790 | WB: 1:1000 |
| β-actin | Cell Signaling  Technology | 3700 | WB: 1:1000 |
| Fibronectin | Proteintech | 15613-1-AP | WB: 1:2000 |
| ZEB1 | Cell Signaling  Technology | 3396 | WB: 1:1000 |
| N-cadherin | Proteintech | 22018-1-AP | WB: 1:2000 |
| E-cadherin | Proteintech | 20874-1-AP | WB: 1:2000 |
| Vimentin | Proteintech | 10366-1-AP | WB: 1:2000 |
| Snail | Abcam | ab216347 | WB: 1:1000 |
| Slug | Cell Signaling  Technology | 9585S | WB: 1:1000 |
| Oct4 | Proteintech | 60242-1-Ig | WB: 1:5000 |
| CD133 | Proteintech | 66666-1-Ig | WB: 1:2000 |
| Nanog | Cell Signaling  Technology | 3580 | WB: 1:1000 |
| ALDH1A1 | Proteintech | 15910-1-AP | WB: 1:1000  IHC: 1:100  IF: 1:100  FC: 0.20 ug per 10^6^ cells in a 100 µl suspension |
| CD44 | Proteintech | 15675-1-AP | WB: 1:2000  IF: 1:100 |
| CD44 | BD Biosciences | 555479 | 20µl for 10^6^ cells |
| CD24 | BD Biosciences | 555427 | 20µl for 10^6^ cells |
| CD8 | Abcam | ab28017 | 10µl for 10^6^ cells |
| TP53 | Proteintech | 60283-2-Ig | WB: 1:5000  IHC: 1:2000 |
| AKT | Proteintech | 60203-2-Ig | WB: 1:5000 |
| P-AKT | Proteintech | 66444-1-Ig | WB: 1:2000 |
| WIP | Santa | sc-390099 | WB: 1:200 |
| YAP | Cell Signaling  Technology | 14074 | WB: 1:1000  IHC: 1:100 |
| PD-L1 | Cell Signaling  Technology | 13684 | WB: 1:1000  IHC: 1:200  FC: 1:200 |
| His | Cell Signaling  Technology | 2365 | WB: 1:1000  IP: 1:25 |
| Flag | Invitrogen | PA1-984B | WB: 1:500 |
| HA | Cell Signaling  Technology | 3724 | WB: 1:1000  IP: 1:50 |
| c-Myc | Proteintech | 10828-1-AP | WB: 1:2000  IF: 1:200 |
| HDAC1 | Proteintech | 10197-1-AP | WB: 1:5000  IF: 1:200 |

**Table S2. Primers and probes sequences used for qRT-PCR ISH and FISH in the experiments.**

| **Name** | **Sense (5'-3’)** | **Antisense (5’-3’)** |
| --- | --- | --- |
| circCFL1 DIV | CTCCAAGGACGCCATCAA | AGAGGGCATAGCGGCAGT |
| circCFL1 CON | CCCTACGCCACCTTTGTC | TTGATCCCTGTCAGCTTCT |
| HDAC1 | ACCGACTGACGGTAGGGACG | CAGAGGGCAGGCAGTGTTTC |
| c-Myc | CGACTCGGTGCAGCCGTATTT | TGTGACCGCAACGTAGGAGGG |
| TP53 | CCTCAGCATCTTATCCGAGTGG | TGGATGGTGGTACAGTCAGAGC |
| U6 | CTCGCTTCGGCAGCACA | AACGCTTCACGAATTTGCGT |
| β-actin | CACTGTGCCCATCTACGAG | AATGTCACGCACGATTTCC |
| GAPDH | GGAGCGAGATCCCTCCAAAAT | GGCTGTTGTCATACTTCTCATGG |
| circCFL1 ISH probe | AGAGGGCATAGCGGCAGT | |
| circCFL1 FISH probe | GATCTCCTTGCCCTCCTCGTAGCAG | |

**Table S3. SiRNAs used for transfection.**

| **Name** | **Sense (5'-3’)** | **Antisense (5’-3’)** |
| --- | --- | --- |
| si-circCFL1-1 | ACTGCTACGAGGAGGGCAATT | TTGCCCTCCTCGTAGCAGTTT |
| si-circCFL1-2 | GAGGAGGGCAAGGAGATCCTT | GGATCTCCTTGCCCTCCTCTT |
| si-HDAC1-1 | CCGGTCATGTCCAAAGTAATT | TTACTTTGGACATGACCGGTT |
| si-HDAC1-2 | GCTCCTCTGACAAACGAATTT | ATTCGTTTGTCAGAGGAGCTT |
| si-c-Myc-1 | GGAACTATGACCTCGACTATT | TAGTCGAGGTCATAGTTCCTT |
| si-c-Myc-2 | GAACACACAACGTCTTGGATT | TCCAAGACGTTGTGTGTTCTT |
| si-TP53-1 | CTACTTCCTGTTTTCTTCGTT | CGTTGTTTTCAGGAAGTAGTT |
| si-TP53-2 | GAAGAAACCACTGGATGGATT | TCCATCCAGTGGTTTCTTCTT |
| si-NC | TTCTCCGAACGTGTCACGTTT | ACGTGACACGTTCGGAGAATT |
